# Ccdc13 is essential for the assembly of ciliary central microtubules

**DOI:** 10.1093/nsr/nwaf095

**Published:** 2025-03-17

**Authors:** Zhimao Wu, Yingying Zhang, Juyuan Liu, Hao Liu, Jin Niu, Yan Li, Shanshan Xie, Xiumin Yan, Xueliang Zhu, Qing Wei

**Affiliations:** School of Synthetic Biology, Shanxi Key Laboratory of Nucleic Acid Biopesticides, Shanxi University, Taiyuan 030006, China; CAS Key Laboratory of Insect Developmental and Evolutionary Biology, CAS Center for Excellence in Molecular Plant Sciences, Chinese Academy of Sciences, Shanghai 200032, China; Center for Energy Metabolism and Reproduction, Institute of Biomedicine and Biotechnology, Shenzhen Institutes of Advanced Technology, Chinese Academy of Sciences (CAS), Shenzhen 518055, China; University of Chinese Academy of Sciences, Beijing 100039, China; State Key Laboratory of Cell Biology, Shanghai Institute of Biochemistry and Cell Biology, Center for Excellence in Molecular Cell Science, Chinese Academy of Sciences, Shanghai 200031, China; University of Chinese Academy of Sciences, Beijing 100039, China; State Key Laboratory of Cell Biology, Shanghai Institute of Biochemistry and Cell Biology, Center for Excellence in Molecular Cell Science, Chinese Academy of Sciences, Shanghai 200031, China; School of Synthetic Biology, Shanxi Key Laboratory of Nucleic Acid Biopesticides, Shanxi University, Taiyuan 030006, China; School of Synthetic Biology, Shanxi Key Laboratory of Nucleic Acid Biopesticides, Shanxi University, Taiyuan 030006, China; Children's Hospital, Zhejiang University School of Medicine, National Clinical Research Center for Child Health, Hangzhou 310052, China; Ministry of Education-Shanghai Key Laboratory of Children's Environmental Health, Institute of Early Life Health, Xinhua Hospital, Shanghai Jiao Tong University School of Medicine, Shanghai 200092, China; State Key Laboratory of Cell Biology, Shanghai Institute of Biochemistry and Cell Biology, Center for Excellence in Molecular Cell Science, Chinese Academy of Sciences, Shanghai 200031, China; School of Synthetic Biology, Shanxi Key Laboratory of Nucleic Acid Biopesticides, Shanxi University, Taiyuan 030006, China

**Keywords:** motile cilia, central microtubules, Ccdc13, Spef1, *Drosophila*

## Abstract

Motile cilia are critical for diverse cellular activities, affecting the survival and development of most eukaryotic organisms. Central microtubules (MTs), which are located in the lumen of ciliary axonemes, are non-centrosomal MTs that are crucial for motile cilia beating. However, the formation mechanism of central MTs remains elusive. Here, by using a *Drosophila* model, we identify Ccdc13 as a novel regulator for the assembly of central MTs. We show that Ccdc13 localizes along the central MTs and is essential for its formation in sperm flagella, with its deletion consequently affecting the sperm motility and the fertility of male flies. Mechanistically, we demonstrated that Ccdc13 directly interacts with Spef1, acting upstream of Spef1 to regulate central MT elongation. Remarkably, we demonstrated that the role of Ccdc13 in ciliary central MT formation is conserved in mammals. *Ccdc13* deficiency in mice leads to the loss of central MTs in motile ependymal cilia, resulting in abnormal cilia motility and hydrocephalus. Our results mark the discovery of Ccdc13 as a novel regulator for ciliary central MT assembly and reveal that the Ccdc13–Spef1 complex is an evolutionarily conserved module that is critical for central MT formation in motile cilia of both flies and mammals.

## INTRODUCTION

Motile cilia/flagella are highly conserved organelles that are found in various organisms, ranging from unicellular protozoa to humans. These structures can beat and have the ability to generate directional fluid flow to drive cell movement or transport materials over cells [1]. In mammals, motile cilia are mainly found in sperms and epithelial cells that line the respiratory tract, the oviduct and brain ventricles, exerting functions such as sperm motility, mucus clearance, ovum transfer and circulation of cerebrospinal fluid [2–5]. Defects in motile cilia result in primary ciliary dyskinesia (PCD) in humans—a rare genetic disorder with symptoms such as chronic rhinosinusitis, chronic bronchitis, infertility and hydrocephalus [6,7].

Motile cilia typically possess a ‘9 + 2’ axoneme structure that consists of nine peripheral microtubule (MT) doublets and a central pair (CP) of MT singlets [8]. The motility of cilia relies on protein complexes that are associated with axonemal MTs [8]. These include outer doublet-associated outer dynein arms (ODAs), inner dynein arms (IDAs), Nexin–Dynein regulatory complexes (N-DRCs) and radial spokes (RS) [1,9–11]. ODAs and IDAs are responsible for generating the main mechanical force required for axoneme beating [12], which is regulated by N-DRCs and the RS [13,14]. The central apparatus (CA), which comprises the CP and numerous associated proteinous projections [15], makes contact with the RS and plays a vital role in maintaining the planar cilia-beating pattern [16–19]. A missing CP causes a shift in the ciliary beat pattern from planar to rotary [4,20,21], resulting in PCD [6,22,23].

The basal body (BB)/centriole serves as a template for the formation of the outer doublet MTs, whereas the CP MTs are non-centrosome MTs and lack a known template for their formation. How CP MTs form remains largely unknown but emerging evidence suggests that a short template is first assembled by initial seeds at the base of the axoneme lumen, followed by elongation and stabilization of the two CP MTs [4]. The formation mechanism of these initial seeds appears to vary across species. In protozoa, self-assembly or γ-tubulin-mediated nucleation is proposed as a mechanism for seed formation [24,25]. In *Drosophila*, the centrosome protein Cep135 has been implicated in CP formation initiation [26–28]. In sperm flagella of *Drosophila*, CP formation starts with the nucleation of a singlet MT within the distal BB lumen during the spermatocyte stage, followed by the assembly of CP during flagella formation in the spermatid stage [27,29]. Cep135 is crucial for singlet MT initiation in the spermatocyte stage. In mammals, a cooperative action between Wdr47, Camsaps and Katanin has been implicated in initiating CP formation [4,30]. Katanin generates MT seeds from peripheral MTs, which are stabilized at their minus ends by Camsaps and then recruited to the central lumen at the ciliary base through Wdr47 [4,31]. However, no pathway has been universally confirmed across species. It remains unclear, for instance, whether γ-tubulin-mediated nucleation plays a role in CP formation in *Drosophila* and mammals. The functional conservation of Wdr47, Camsaps and Katanin in *Drosophila* and protozoa is also not fully understood and there is no evidence to suggest that Cep135 plays a role in CP formation in mammals. Therefore, the initiation mechanism for CP MT formation is likely to vary across species. Furthermore, the mechanism that underlies the CP elongation remains largely unknown. Spef1 (Sperm Flagellar 1), also called CLAMP (CaLponin-Homology and MT-Associated Protein) [32–34], is considered a critical protein that is involved in the elongation of CP MTs [21]. Spef1 is an evolutionarily conserved MT-bundling protein that possesses an MT-binding calponin-homology (CH) domain at its N-terminus and a coil-coiled (CC) region at its C-terminus [21]. Recent studies have shown that Spef1 is associated with the CP MT seam, where it may crosslink and stabilize the CP MTs [35]. However, it is still uncertain whether Spef1 plays a similar role in CP formation in invertebrates. The evolutionary conservation and mechanisms of CP MT formation remain largely unclear.

*Drosophila* sperm flagella provide an excellent model for studying CP MT formation due to the well-understood processes involved in flagellar development. In *Drosophila*, sperm flagella formation begins in early spermatocytes and can originate from both mother and daughter centrioles. In the initial stage, during the stage of early spermatocytes, the V-shaped centriole pair recruits transition fiber and transition zone proteins, docks to the plasma membrane and forms a short primary cilia-like cap structure [36,37]. As meiosis progresses, the primary cilia are internalized along with the BB, which is critical for spindle assembly [38]. Following meiosis, the ciliary cap migrates away from the BB and the flagella elongation begins [36]. Interestingly, it has been demonstrated that the CP formation is initiated during the spermatocyte stage, in which a singlet central MT is nucleated within the distal BB lumen. As flagellar elongation proceeds during the spermatid stage, an additional central MT assembles, resulting in the formation of the CP. To date, Cep135 is the only protein confirmed to be involved in CP formation in *Drosophila*, so much remains to be understood about the full molecular mechanisms that underlie CP formation.

In this study, we initially aimed to understand the function of Ccdc13 in cilia formation by using a *Drosophila* model. Unexpectedly, we found that Ccdc13 is specifically required for CP MT formation in sperm flagella. Subsequent studies revealed that Ccdc13 localizes on CP, where it interacts with Spef1 to facilitate CP MT assembly. Interestingly, we furthermore demonstrated that the role of Ccdc13 in CP MT formation is conserved in mammals, as *Ccdc13* deficiency in mice also leads to CP loss. Therefore, we have identified Ccdc13 as a newly discovered CP protein that is essential for CP MT formation in both *Drosophila* and mammals.

## RESULTS

### *Drosophila* Ccdc13 is essential for sperm motility

**Figure 1.**
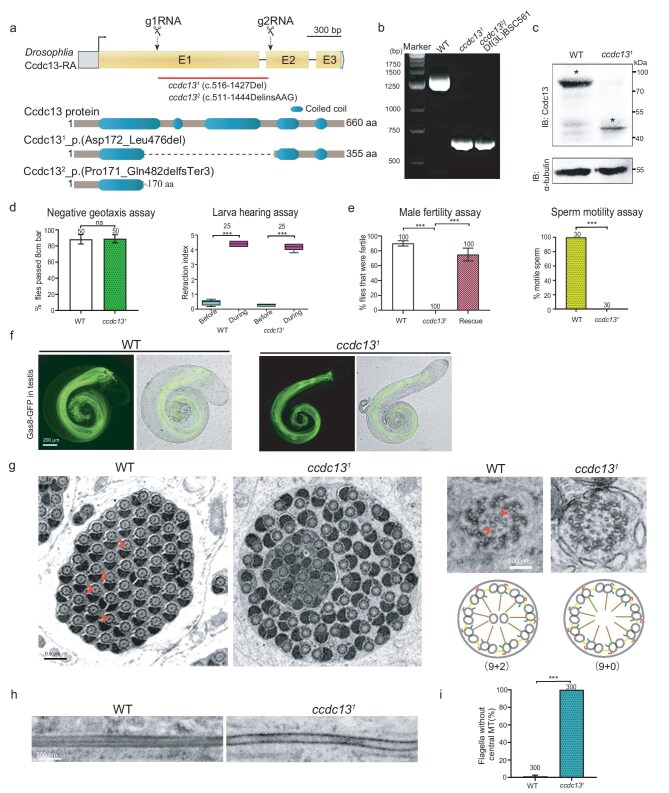
Ccdc13 is essential for CP MT formation in *Drosophila*. (a) Generation of *Ccdc13* deletion mutant (*ccdc13^1^*) flies. Schematics display the genomic structure (upper panel) and protein structure (lower panel) of Ccdc13. The gRNA target sites are shown. *Ccdc13^1^* has a deletion in its cDNA from nt 516 to 1427, leading to an in-frame deletion of 305 aa (aa 172 to 476) from Ccdc13. *Ccdc13^2^* mutant has a deletion in cDNA from nt 511 to 1444, resulting in a reading-frame shift. (b) Genotyping of the *ccdc13^1^* mutant and *ccdc13^2^* mutant using PCR. The PCR products are 1980 bp long for wild type (WT) and 1065 bp long for the mutant. (c) Western blot analysis of the testes showed the presence of a truncated protein product of Ccdc13 in the *Ccdc13^1^* mutant. α-tubulin was used as the loading control. The asterisk indicates the target band. (d) *Ccdc13^1^* mutant flies had normal negative geotaxis and hearing. (e) *Ccdc13^1^* males were infertile due to the immobility of their sperms. Note that the infertility defect was rescued by expressing GFP–Ccdc13. (f) The sperm flagella in *ccdc13^1^* testes showed no defects in elongation compared with those in WT testes. The Nexin–Dynein regulatory complex (N-DRC) protein Gas8 was used to label flagella. (g, h) *ccdc13^1^* flagella lacked the CP. In electron microscopy (EM) images of testis (g) cross sections and (h) longitudinal sections, both WT and *ccdc13^1^* mutants had 64 spermatids in each cyst. The arrowheads denote representative central MTs in the WT cross sections; the central MT was completely lost in *ccdc13^1^* mutants. The quantification data (i) were obtained from >300 flagella in three flies. Scale bars: 200 μm (f), 0.5 μm (g, full-scale images), 100 nm (g, zoomed-in areas). Quantification results are presented as mean ± SD, unless stated otherwise. Student's *t*-test: ns, no significance; ***P < 0.001.

Although Ccdc13 is implicated in ciliogenesis [39], its molecular function remains poorly understood. Phylogenetic analysis suggested that potential orthologs of Ccdc13 exist in species that possess motile cilia (Fig. S1), implying a conserved role in motile cilia throughout the evolution. To elucidate the molecular function of Ccdc13 by using a *Drosophila* model, we performed a protein homology search by using BLAST and identified CG13032 as the sole *Drosophila* homolog of mammalian Ccdc13 (Fig. S2). We then used the CRISPR-Cas9 technique to generate a Ccdc13 deletion mutant and obtained two mutant alleles: *ccdc13^1^* (c.516–1427Del) and *ccdc13^2^* (c.511–1444DelinsAAG). *ccdc13^1^* is predicted to be an in-frame deletion, leading to a mutant Ccdc13 that lacks the middle segment (aa 172–476) (Fig. 1a and b). Western blot analysis confirmed that the full length of Ccdc13 was absent in *ccdc13^1^* mutant flies (Fig. 1c). *ccdc13^2^* is predicted to cause a frameshift that results in a truncation with only the N-terminal 170 amino acids remaining (Fig. 1a and b).

*ccdc13^1^* mutants were viable and exhibited normal walking and flying abilities (Fig. 1d). No obvious defects were observed in sensory neuron cilia-related behaviors, such as climbing and hearing (Fig. 1d). However, we observed that the males were completely infertile—a phenotype that was effectively rescued by the expression of exogenous Ccdc13 (Fig. 1e). Further examination revealed that the mutation resulted in a severe defect in sperm motility (Fig. 1e), explaining the infertility that was observed in the flies.

The homozygous *ccdc13^2^* mutant flies failed to eclose, for unknown reasons. However, when crossed with the chromosome deficiency line Df(3L)BSC561, which carries a deletion on chromosome 3L that spans the 73A2–73C1 region, including the *Ccdc13* gene, *ccdc13^2^*/*Df(3L)BSC561* flies were viable. As this deficiency removes *Ccdc13, ccdc13^2^*/*Df(3L)BSC561* flies essentially represent a null mutant for *Ccdc13*. Interestingly, like *ccdc13^1^*, these flies showed no obvious behavioral defects, but males were infertile. Due to the similar phenotype and easier genetic manipulation, we chose the *ccdc13^1^* mutant for our subsequent studies.

### CP MTs were lost in *ccdc13^1^* sperm flagella

We examined the testes and observed normal morphology and size of testes in *ccdc13^1^* mutants with normally elongated sperm cysts (Fig. 1f). We thus reasoned that Ccdc13 might affect the motility-related structures of sperm flagella. Immunofluorescence examination of the localization of the ODA component CG6971/Dnali1, IDA component CG1571/Dnai2, RS component Rsph1 and N-DRC component Gas8 indicated that they all normally localized on sperm flagellar axoneme in *ccdc13^1^* mutants (Fig. S3). Interestingly, our transmission electron microscopy (TEM) analysis of the flagella ultrastructure revealed a complete loss of the CP in *ccdc13^1^* mutants, whereas the number of flagella and the organization of the doublet MTs were overall normal (Fig. 1g–i). Therefore, *Drosophila* Ccdc13 is essential for CP formation.

### Ccdc13 is a CP-associated protein

Given the importance of Ccdc13 in CP formation, we were curious as to whether it is associated with CP. To investigate this, we generated transgenic flies that expressed GFP–Ccdc13 driven by the ubiquitin promoter and analysed its subcellular localization.

**Figure 2.**
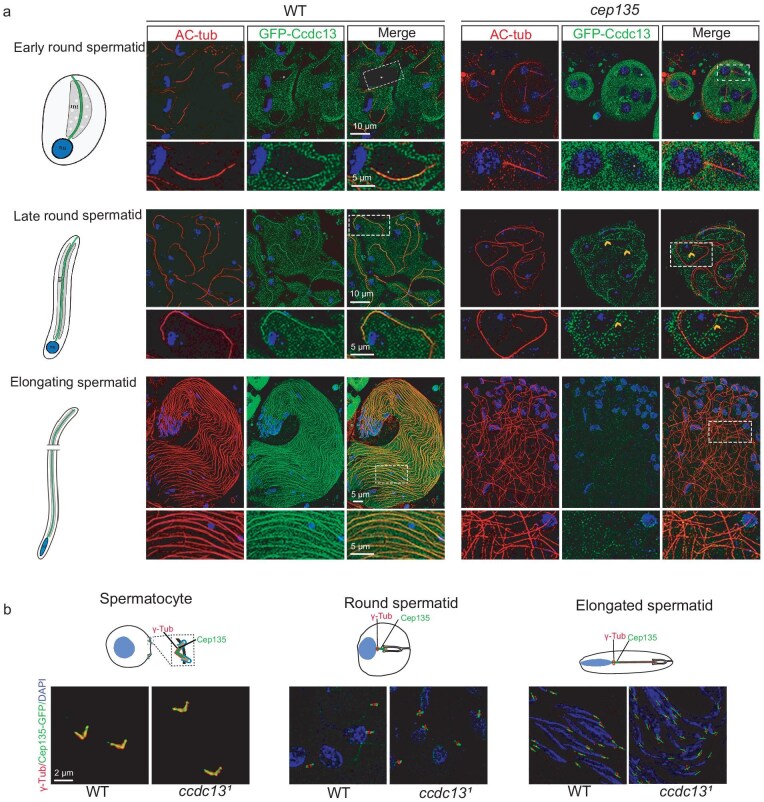
Ccdc13 localizes to the CP in *Drosophila*. (a) Ccdc13 displayed CP-dependent axonemal localization. In WT testes, Ccdc13 first appeared along the axoneme of the flagella in early-round spermatids and the localization persisted in late-round spermatids and elongating spermatids. In testes of *cep135* mutants, whose flagella lacked the CP, Ccdc13 no longer displayed axonemal localization. The flagella were labeled by using AC-tubulin (red). The white asterisk indicates the leaf-blade-shaped mitochondria region in early-round spermatocytes. The arrowhead represents the microtubule-like GFP–Ccdc13 signal in the cytosol of the *cep135* mutant. (b) Ccdc13 was not required for the basal body localization of Cep135. The basal body was labeled by using γ-tubulin (red).

Live imaging of GFP–Ccdc13 in testes revealed that Ccdc13 was not present in cilia in spermatocytes but was specifically localized to the flagella of spermatids (Fig. S4A and B). Notably, the intensity of the GFP–Ccdc13 was lower in the MT growth zone at the tail end of the flagella than in the MT stable zone (Fig. S4B). Further investigation revealed that Ccdc13 gradually appeared on the spermatid axoneme in early-round spermatids as the axoneme initiated elongation (Fig. 2a). In late-round spermatids and elongating spermatids, as the sperm axoneme extended farther, Ccdc13 was distributed along the entire sperm axoneme (Fig. 2a).

CP formation has been reported to be defective in *cep135*-deficient *Drosophila* [26,27]. To determine whether Ccdc13 is associated with CP, we examined the GFP–Ccdc13 signal in *cep135* mutants and observed the absence of GFP–Ccdc13 in their sperm flagella (Fig. 2a). Additionally, we analysed Cep135 localization in *ccdc13* mutants and found that it remains normal in both spermatocytes and spermatids (Fig. 2b). Therefore, Ccdc13 is a CP-localized protein that functions downstream of Cep135.

### Ccdc13 interacts with Spef1 in *Drosophila*

**Figure 3.**
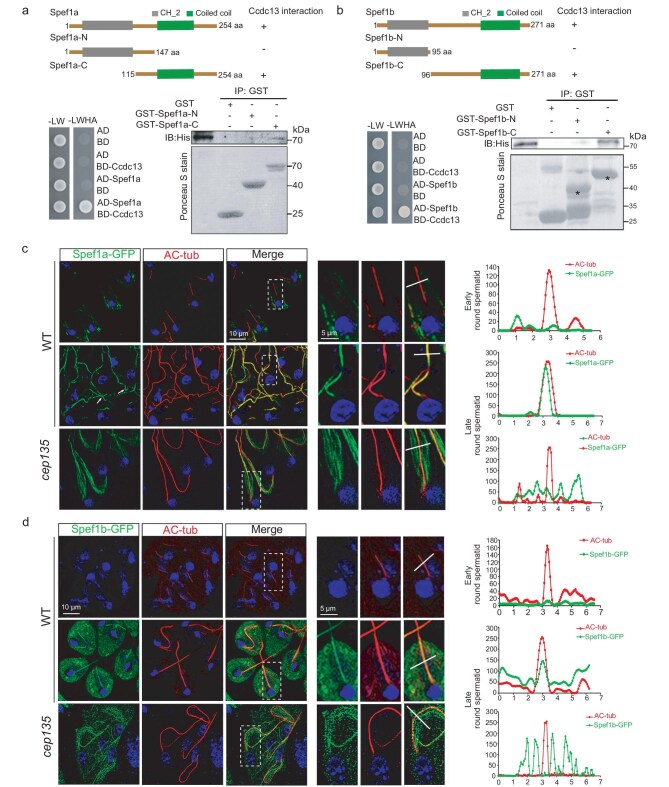
Ccdc13 interacts with CP protein Spef1 in *Drosophila*. (a) Ccdc13 exhibited direct interaction with Spef1a in both the Y2H assay (lower left panel) and the GST pull-down assay (lower right). The upper panel displays the schematics of the Spef1a constructs. In the Y2H assay, the interaction of Ccdc13 with Spef1a was evidenced by colony growth on the SD–Ade–Leu–Trp–His plates. In the GST pull-down assay, Ccdc13 directly interacted with the C-terminal of Spef1a whereas no interaction was observed with its N-terminal. L: Leu, W: Trp, H: His, A: Ade. (b) Ccdc13 exhibited direct interaction with Spef1b in both the Y2H assay (lower left panel) and the GST pull-down assay (lower right). The upper panel displays the schematics of the Spef1b constructs. In the Y2H assay, the interaction of Ccdc13 with Spef1b was evidenced by colony growth on the SD–Ade–Leu–Trp–His plates. In the GST pull-down assay, Ccdc13 directly interacted with the C-terminal of Spef1b whereas no interaction was observed with its N-terminal. L: Leu, W: Trp, H: His, A: Ade. (c) Subcellular localizations of Spef1a during spermiogenesis. In early-round spermatids, Spef1a was not observed on the axonemes of the flagella. In late-round spermatids, Spef1a began to appear on the axonemes of the flagella. In *cep135* mutants, the Spef1a signal on the axoneme was notably impaired, exhibiting abnormal signal distribution in the cytosol. The right panels depict zoomed-in views of the axonemes (white dotted rectangles) to highlight specific details. Line scans were performed to show correlations between axonemes and Spef1a. (d) Spef1b–GFP localizes to the sperm flagella starting from late-round spermatids and this localization relies on Cep135.

To uncover how Ccdc13 contributes to CP formation, we conducted a screening for Ccdc13-interacting proteins in testes by using yeast two-hybrid assays. For this purpose, a cDNA library that was derived from *Drosophila* testes was used and our screening yielded 11 candidate genes, including two encoding *Drosophila* homologs of Spef1 (Fig. 3a and b, and Table S2). Given that Spef1 has been shown to play a critical role in CP formation in mammals [21], we hypothesized that *Drosophila* Spef1 might also be involved in CP formation, leading us to focus on these two candidates for further characterization.

In the *Drosophila* genome, the two homologous genes of *Spef1* are known as *CG12395* and *CG16719*, which we refer to as *Spef1a* and *Spef1b*, respectively. *Spef1a* is situated on the X chromosome whereas *Spef1b* is located on the 3rd chromosome. A sequence alignment analysis showed that the encoded proteins, Spef1a and Spef1b, share only ∼45% similarity (Fig. S5A and B). Notably, Spef1a exhibited a higher degree of similarity to human Spef1 compared with Spef1b (Fig. S5B).

To validate the physical interaction between Ccdc13 and Spef1, we conducted glutathione-S-transferase (GST) pull-down assays and found that Ccdc13 indeed interacts with both Spef1a and Spef1b. Both Spef1a and Spef1b comprise an N-terminal MT-binding CH domain and a C-terminal CC domain. Notably, Ccdc13 exhibited a specific interaction with their C-terminal CC domain, while showing no interaction with their N-terminal CH domain (Fig. 3a and b).

### Conserved role of *Drosophila* Spef1a in CP formation

Next, we investigated the subcellular localization of Spef1a and Spef1b. By utilizing transgenic flies that were expressing Spef1a–GFP or Spef1b–GFP under the ubiquitin promoter, we observed robust colocalization of both proteins with the axoneme marker Ac-tub on sperm flagella in wild type flies. However, this colocalization was significantly compromised in *cep135* mutants, showing abnormal cytosolic MT-like signals that surrounded the axoneme (Fig. 3c and d). As Spef1a is known to directly bind MTs through its N-terminal CH domain, we propose that, in *cep135* mutants, the absence of central MTs in sperm flagella leads to an accumulation of Spef1a on cytosolic MTs. The loss of Spef1a–GFP and Spef1b–GFP signals from sperm flagella in *cep135* mutants indicates that they are indeed localized to the CP. It is noteworthy that the presence of Spef1a and Spef1b on flagella occurs slightly later than that of Ccdc13. Ccdc13 is evident in the early-round spermatids stage (Fig. 2a) whereas Spef1a and Spef1b emerge at a later stage (Fig. 3c and d). This timing difference was further confirmed by co-staining GFP–Ccdc13 and Spef1b–tdTomato, as shown in Fig. S6, in early-round spermatids, which showed that Ccdc13 is present along the flagella whereas Spef1b remains undetectable.

**Figure 4.**
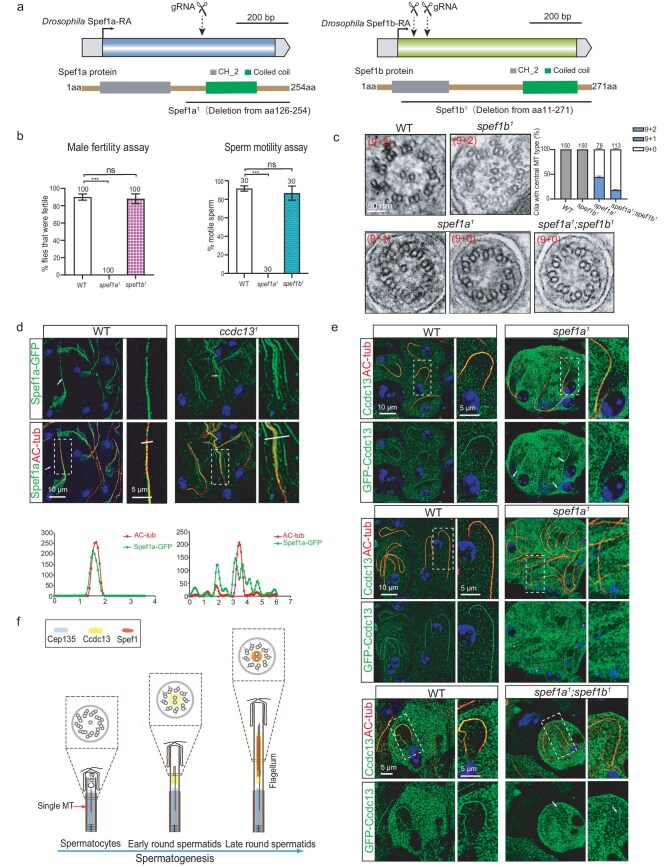
Ccdc13 acts upstream of Spef1a in CP MT formation. (a) Generation of *Spef1a* and *Spef1b* mutant flies. Schematics display the genomic (upper panel) and protein (lower panel) structures of Spef1a and Spef1b. The gRNA target sites are shown. *spef1a^1^* had a deletion in cDNA from nt 376 to 382, resulting in a frameshift and deletion of the C-terminal half of the protein (aa 126 to the end at 254). *spef1b^1^* had a deletion in cDNA from nt 31 to 136, introducing a stop codon and resulting in the deletion of almost the whole protein (leaving only 10 aa). (b) *spef1a* was essential for male fertility and sperm motility. Quantification results are presented as mean ± SD. Student's *t*-test: ns, no significance; ***P < 0.001. (c) Representative EM images of flagellar cross sections from wild type (WT), *spef1b^1^, spef1a^1^* and *spef1a^1^; spef1b^1^* fly mutants. The quantification results are presented on the right. (d) Spef1a failed to colocalize with the axoneme in *ccdc13^1^* flagella. Arrows indicate typical flagella. Axonemes in dotted rectangles are zoomed-in in the right-hand panels to show details. Line scans were performed to show correlations between axoneme and Spef1a. (e) Axonemal localization of Ccdc13 was detected in only early-round spermatids of *spef1a^1^* flies and *spef1a^1^; spef1b^1^* double-mutant flies, albeit with a compromised signal compared with that in WT flies. Arrows and asterisks, respectively, indicate typical Ccdc13-positive and Ccdc13-negative flagella in *spef1a^1^* and *spef1a^1^; spef1b^1^* testes. Axonemes in white dotted rectangles are zoomed-in in the right-hand panels to show details. (f) A model for the role of Ccdc13 and Spef1a in the CP MT formation in sperm flagella of *Drosophila*. In spermatocytes, Cep135 initiates the formation of a singlet central MT. In early-round spermatids, Ccdc13 emerges on the CP, potentially stabilizes the singlet central MT and/or initiates the formation of the second central MT. Following this, Spef1a is recruited and, together with Ccdc13, promotes the elongation and stabilization of CP MTs.

To clarify the role of Spef1 in *Drosophila*, we obtained *spef1a^1^* (c.376–383Del) and *spef1b^1^* (c.31–136DelinsAACTCC) deletion mutants by using the CRISPR-Cas9 technique (Fig. 4a and Fig. S7A and B). In the *spef1a^1^* mutant, the C-terminal half of Spef1a was lost due to a frameshift that was caused by the mutation. In the *spef1b^1^* mutant, almost the entire Spef1b (aa 11 to the end at 271) was lost due to a frameshift that was caused by the deletion. Both *spef1a^1^* and *spef1b^1^* flies were viable and did not exhibit apparent defects in cilia-related sensory behaviors (Fig. S8A and B). Interestingly, the *spef1a^1^* males were infertile and their sperms were immotile (Fig. 4b). In contrast, the loss of Spef1b did not affect male fertility or sperm motility (Fig. 4b). After examining the ultrastructure of sperm flagella by using TEM, we found that the *spef1b^1^* mutants exhibited normal CP formation whereas the *spef1a^1^* mutants showed either the loss of both CP MTs (55.8%) or the loss of one CP MT (44.2%) in the sperm axoneme (Fig. 4c and Fig. S8C). These findings suggest that Spef1a plays a crucial role in CP formation in *Drosophila*. To investigate the potential functional redundancy between Spef1a and Spef1b in CP formation, we generated *spef1a; spef1b* double mutants, Interestingly, we found that CP MTs were almost completely absent in the double mutants (Fig. 4c and Fig. S8C), indicating that Spef1b has a redundant role in CP formation in the absence of Spef1a.

Next, we investigated the relationship between Ccdc13 and Spef1a. In *ccdc13* mutants, Spef1a–GFP was lost from the axoneme, which instead showed a MT-like signal in the cytosol between the axoneme and the flagellar membrane (Fig. 4d). In *Spef1a^1^* mutants, the axonemal localization signal of GFP–Ccdc13 was significantly diminished at the late-spermatid stage (Fig. 4e). However, a reduced but still detectable Ccdc13 signal was observed on axonemes in early-round spermatids (Fig. 4e). Notably, the remaining axonemal Ccdc13 signal was still detectable in *spef1a; spef1b* double mutants (Fig. 4e), possibly because Ccdc13 appears on the axoneme slightly earlier than Spef1a/b (Figs 2a and 3c and d).

Taken together, our studies, through using the *Drosophila* model, demonstrated that Ccdc13–Spef1 is a key complex for CP formation. We propose that the CP formation in the sperm flagella of *Drosophila* is initiated in the spermatocyte stage, in which a singlet central MT is nucleated with the involvement of Cep135 [27]. In the early-round spermatid stage, Ccdc13 is initially recruited, potentially stabling the singlet central MT and/or initiating the formation of the second central MT. Subsequently, Spef1 is recruited to facilitate the CP elongation and stabilization (Fig. 4f).

### Mammalian Ccdc13 localizes to CP of motile cilia and is required for their planar beat pattern

**Figure 5.**
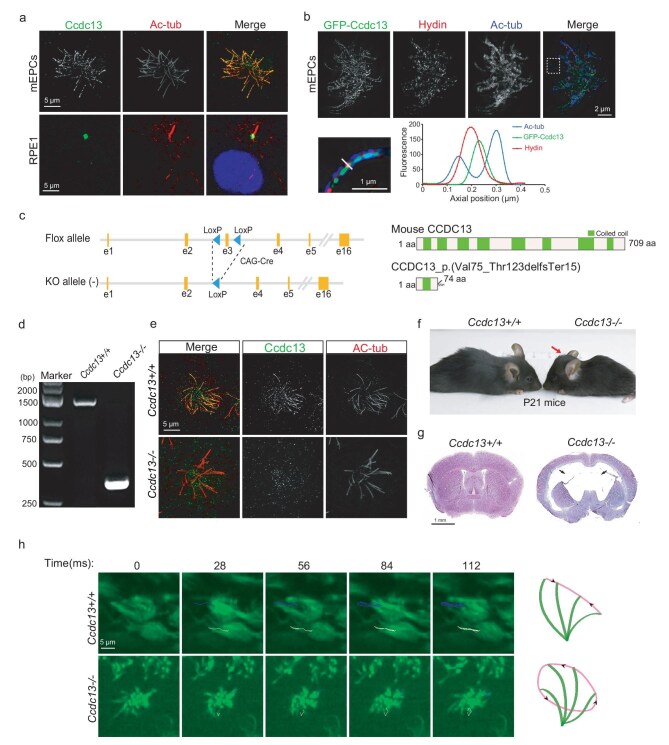
Mammalian Ccdc13 is an important CP protein whose depletion in mice results in hydrocephalus. (a) Ccdc13 specifically localized to motile cilia but not primary cilia in mammalian cells. Cultured mEPCs (top panel) were fixed at Day 15 post serum starvation whereas RPE1 cells (bottom panel) were fixed 24 h post serum starvation. Representative confocal images are presented. (b) Ccdc13 colocalized with Hydin (arrows) in the axoneme lumen of mEPC cilia. Cultured mEPCs were infected with lentivirus to express GPF–Ccdc13 and fixed at Day 7. Hydin served as a CP marker. Representative 3D-SIM images are presented. The framed region is magnified to show details. (c) The strategy for generating *Ccdc13* knockout (KO) mice. A *Ccdc13 flox* allele was generated with two loxP sites flanking Exon 3 (e3). Upon crossing with Cre mice, this was expected to lead to the deletion of Exon 3, causing a frameshift and retaining only 74 amino acids of the N-terminal. (d) Genotyping of *Ccdc13^−/−^* mutant mice using PCR. The PCR products were 1532 bp long for WT and 403 bp long for *Ccdc13^−/−^* mice. (e) Anti-Ccdc13 staining confirmed the absence of Ccdc13 in *Ccdc13^−/−^* multicilia. mEPCs at Day 15 were fixed, followed by immunostaining. (f) *Ccdc13^−/−^* mice displayed a dome-shaped skull (arrow). (g) *Ccdc13^−/−^* mice exhibited hydrocephalus. Representative HE-stained coronal sections of the brain are presented. Arrows indicate enlarged ventricles. (h) Typical image sequences of ependymal cilia were acquired at 200 frames per second (fps) by using living brain slices of P21 mice of indicated genotypes. Trajectories of cilia are shown in each sample.

To understand whether the role of Ccdc13 in CP formation is conserved in mammals, we first examined its subcellular localization in the cilia of mammalian cells. We observed that both endogenous mouse Ccdc13 and exogenous GFP-tagged mouse Ccdc13 (GFP–Ccdc13) were localized to the motile cilia of multiciliated mouse ependymal cells (mEPCs) (Fig. 5a and Fig. S9). In contrast, Ccdc13 was detected at the BB region but not in the primary cilia of human retinal pigment epithelium (hTERT-RPE1) cells (Fig. 5a). Furthermore, when Hydin and acetylated tubulin were respectively used as a CP marker and an axoneme marker, super-resolution 3D structured illumination microscopy (3D-SIM) clearly revealed the localization of Ccdc13 in the axonemal central lumen (Fig. 5b). Therefore, Ccdc13 is also a CP-associated protein in mammals.

Next, we generated *Ccdc13* knockout mice by crossing *Ccdc13^Flox/Flox^* mice with *Cag–Cre* mice [40], resulting in a deletion of Exon 3, predicting a frameshift and leaving only 74 amino acids at the N-terminal (Fig. 5c and d). Immunofluorescence staining showed complete loss of Ccdc13 from the axonemes of motile cilia in *Ccdc13^−/−^* mEPCs (Day 15) (Fig. 5e).

Although *Ccdc13^−/−^* mice were born normally, most of them died at 2–4 weeks old due to severe hydrocephalus, evidenced by dome-shaped heads and abnormally enlarged ventricles (Fig. 5f and g). Given that hydrocephalus can be induced by the immobility of ependymal cilia [41], we examined the motilities of ependymal cilia through live imaging of the brain ventricles. While ependymal cilia in the control mice displayed regular coordinated planar beating, those in the *Ccdc13^−/−^* mice exhibited uncoordinated and rotatory beating (Fig. 5h and Supplementary Movies S1–S3), which is typically observed in animals with CP defects [4].

### Mammalian Ccdc13 is required for the CP formation

**Figure 6.**
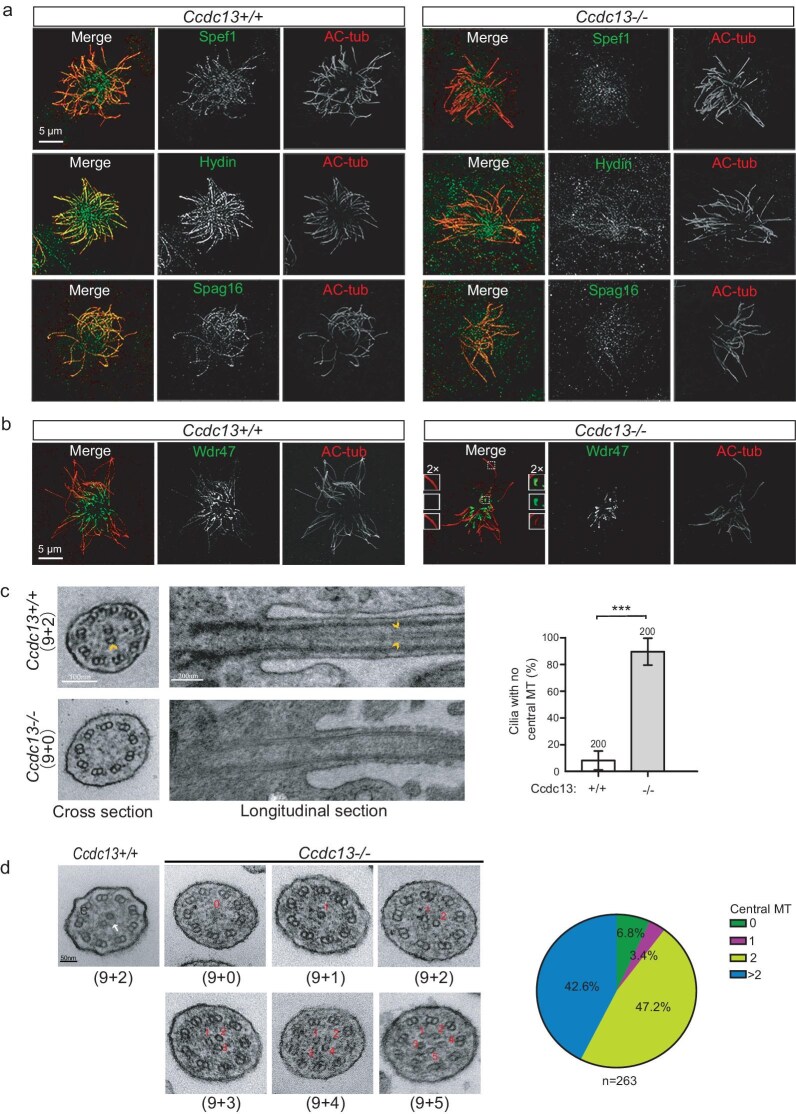
Mammalian Ccdc13 is critical for CP formation. (a) CP-associated proteins Spef1, Hydin and Spag16 failed to show prominent axonemal localizations in *Ccdc13^−/−^* mEPCs. Cultured mEPCs were fixed at Day 15. (b) The localization of Wdr47 was mildly affected in short cilia but almost lost in long cilia in *Ccdc13^−/−^*. (c) *Ccdc13^−/−^* ependymal multicilia lacked a CP. Representative TEM images from ependymal tissues of P21 mice are presented. Arrowheads indicated central MTs. Quantification results were obtained from three pairs of littermates, with 200 cross sections of cilia scored for each group of mice. (d) Ccdc13 defect impairs the CP formation of respiratory multicilia. Tracheal epithelial tissues of P21 mice were fixed and processed for TEM to observe CP MT (arrowheads) formation. The pie chart summarizes the percentages of the axoneme section with different central MT numbers.

To determine whether the deletion of Ccdc13 results in defective CP formation in mice, we first examined the localization of known CP proteins in Day-15 mEPCs of *Ccdc13^−/−^* mice. As expected, Spef1 was no longer located in the cilia of *Ccdc13^−/−^* mEPCs (Fig. 6a). In addition, ciliary localizations of Hydin—a protein that is associated with the C2 MT [42,43] and Spag16, which localizes to the bridge between C1 and C2 [44]—were also markedly reduced in C*cdc13^−/−^* mEPCs (Fig. 6a). These results suggest a loss of the CP in the cilia of C*cdc13^−/−^* mEPCs. However, we observed that Wdr47—the critical factor for CP-assembly initiation [4]—could still localize to the proximal region of the cilia in C*cdc13^−/−^* mEPCs, although its signal was almost absent in the upper segment (Fig. 6b). This suggests that Ccdc13 may not be involved in the initiation of CP assembly, but rather in its elongation and maintenance.

Subsequently, we performed TEM on brain ependymal cilia and found that the CP was lost in 90% of the cilia of *ccdc13^−/−^* cells (Fig. 6c), indicating that Ccdc13 is indeed critical for CP formation in mammals. Additionally, we performed TEM analysis on the respiratory cilia of Ccdc13 global knockout mice and observed three distinct phenotypes (Fig. 6d): (i) a small percentage of cilia either lacked central MTs or had a single central MT, indicating that Ccdc13 is also involved in CP formation in tracheal cilia; (ii) about half of the cilia exhibited two central MTs, but these appeared unbundled compared with wild-type, with associated structures missing, suggesting abnormalities; (iii) about 40% of the cilia contained more than two central MTs, suggesting that Ccdc13 may have a tissue-specific role in regulating the number of central MTs. These results suggest that Ccdc13 is not only crucial for CP MT formation, but also has a tissue-specific function in controlling the number of central MTs.

### Mammalian Ccdc13 interacts with Spef1

**Figure 7.**
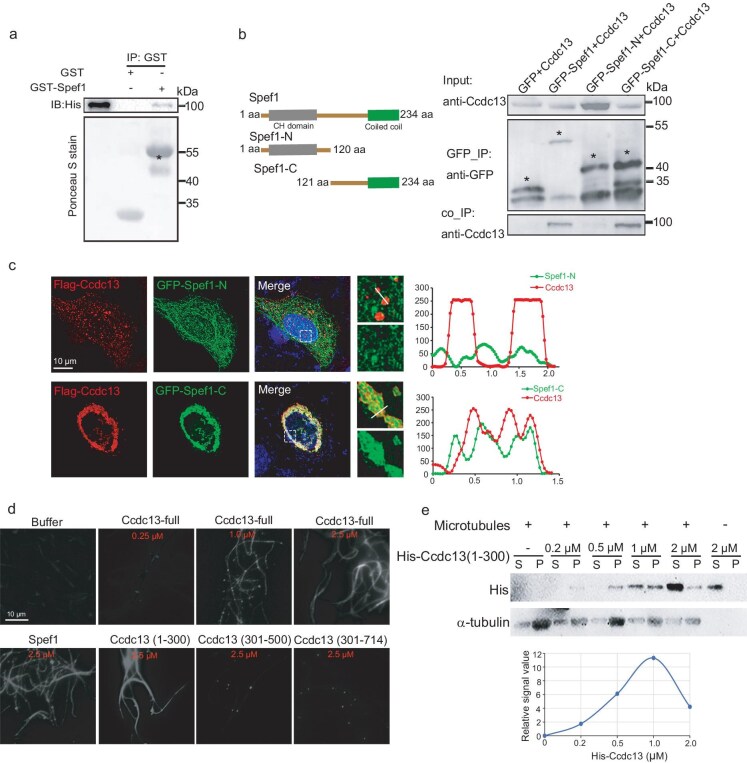
Mammalian Ccdc13 interacts with Spef1 and exhibits MT-binding activity. (a) Mouse Ccdc13 directly interacted with mouse Spef1 in GST pull-down assays. Asterisks indicate the position of GST–Ccdc13. (b) Mouse Ccdc13 associated with the C-terminus of mouse Spef1 *in vivo*. The indicated proteins were transiently expressed in HEK293T cells, followed by co-immunoprecipitation (Co-IP) assays. Asterisks indicate the position of GFP–Spef1 and its truncations. (c) *In vivo* colocalization of Ccdc13 and the C-terminal of Spef1 in mammalian cells. The colocalization of Spef1-C with Ccdc13 was observed whereas there was no colocalization between Ccdc13 and Spef1-N. (d) Ccdc13 exhibited the ability to bundle MTs, with the N-terminal amino acids 1–300 as the region responsible for this activity. MTs that were polymerized *in vitro* were incubated with the specified concentrations of the respective proteins and subsequently imaged. (e) Ccdc13 (1–300) effectively co-sedimented with MT pellets in the MT-pelleting assay. P, pellet fraction containing Taxol-stabilized MTs; S, supernatant fraction containing soluble proteins. Results are representative of two independent experiments.

Next, we wondered whether the interaction between Ccdc13 and Spef1 is conserved in mammals. By using *in vitro* GST pull-down and *in vivo* co-immunoprecipitation assays, we confirmed that mammalian Ccdc13 also interacted with Spef1 (Fig. 7a and b). Furthermore, we demonstrated that Ccdc13 specifically co-immunoprecipitated with the Spef1 C-terminal CC domain, but not its N-terminal CH domain, indicating that mammalian Ccdc13, similarly to its *Drosophila* homolog, interacts with the Spef1 CC domain. Consistently with these biochemical results, the immunofluorescence assay showed robust colocalization between Ccdc13 and the CC domain of Spef1 when exogenously expressed in mammalian cells, but no colocalization was observed between Ccdc13 and the CH domain of Spef1 (Fig. 7c).

### Ccdc13 binds and bundles MTs

Given the critical role of Ccdc13 in CP MT formation and its close association with MT-bundling Spef1, we wondered whether Ccdc13 also possesses MT-binding or -bundling abilities. To this end, we performed *in vitro* MT-bundling assays. As a positive control, Spef1 significantly enhanced the MT bundling in our assay (Fig. 7d). Interestingly, we observed that purified His-Ccdc13, at various concentrations, induces MT bundling in a concentration-dependent manner (Fig. 7d). Moreover, we pinpointed the N-terminal 1–300 amino acids of Ccdc13 as the region that was responsible for this activity, while its C-terminal region showed no response in the MT-bundling assay. Next, we conducted the MT pellet assay and found that the Ccdc13 (1–300) effectively co-sedimented with the MT pellet fraction (Fig. 7e), indicating that Ccdc13 (1–300) could bind to MTs. As a result, Ccdc13 has emerged as a novel MT-associated protein with the capacity to stabilize and bundle MTs.

## DISCUSSION

CP MTs are critical for the proper beating of motile cilia, yet the mechanisms that underlie their formation remain poorly understood. In this study, we identified Ccdc13 as an evolutionarily conserved key regulator of CP MT assembly. We demonstrated that Ccdc13 localizes to the CP in both *Drosophila* and mice. Disruption of Ccdc13 in *Drosophila* results in the complete loss of CP MTs in sperm flagella, leading to male infertility due to immotile sperm. Similarly, knockout of Ccdc13 in mice causes defects in the CP of brain ependymal cilia, resulting in abnormal ciliary beating and hydrocephalus. Additionally, we showed that Ccdc13 directly interacts with the MT seam-binding protein Spef1 in both *Drosophila* and mammals. While the role of Spef1 in mammalian CP formation has been previously documented [21], we further validated its conserved function in *Drosophila* CP assembly. Taken together, our findings uncover an evolutionarily conserved Ccdc13–Spef1 complex that is crucial for CP MT formation across species.

As a type of non-centrosomal MT, the assembly of CP MTs lacks templates. Their formation involves initiation by tubulin seeds, followed by elongation and stability maintenance [4]. In *Drosophila*, CP MT formation initiates at the spermatocyte stages with the formation of a single central MT [27]. Given that Ccdc13 emerges on a CP from the early-round spermatid stages when paired MTs begin to emerge, it is conceivable that Ccdc13 is not involved in the initiation of CP MT singlets, but rather functions in CP MT elongation and/or maintenance. As Ccdc13 appears slightly earlier than Spef1a/b, we propose that Ccdc13 may stabilize the singlet central MT and/or initiate the formation of the second central MT. Following this, Spef1a is recruited and, together with Ccdc13, they collaborate to facilitate the elongation and stabilization of CP MTs. A recent study has demonstrated that Spef1 binds specifically to the MT seam in both C1 and C2 and stabilizes MTs [35]. Interestingly, we also revealed that Ccdc13 has the ability to bind and promote MT bundles. The molecular mechanism by which Ccdc13 and Spef1 promote CP MT assembly and maintenance requires further investigation. Future exploration of the functions and interactions of Ccdc13 and Spef1 by using *Drosophila* and mammalian models will provide valuable insights into the mechanisms that govern the elongation and stability of CP MT formation.

CP MTs are conserved structures in motile cilia, but the mechanisms of their formation likely vary significantly across species. As we highlighted in the introduction, the initiation mechanism of CP MT assembly varies across species. Our findings in both *Drosophila* and mammals underscore the conservation of the Ccdc13–Spef1 module in CP elongation and stability, which highlights the significance of our discovery and its potential broader implications for understanding CP assembly. However, our study also reveals a fascinating complexity: unlike in motile mEPC cilia, the loss of Ccdc13 in tracheal cilia leads to the formation of additional central MT singlets, suggesting that variations in the mechanisms of CP MT formation or dynamics can occur even within the same organism. We hypothesize that these differences may be related to structural diversity. The axonemes of motile epithelial cilia (mEPCs) and respiratory cilia exhibit notable structural diversity, as recently demonstrated by Leung *et al.* [45]. Therefore, an understanding of how Ccdc13 and Spef1 interact with species-specific proteins and other regulatory factors in various ciliated cells is crucial for comprehending the diversity of CP-assembly mechanisms.

Our study has important clinical implications, particularly in understanding the ciliopathies associated with defects in motile cilia. Impairments in the CP of motile cilia can lead to various severe conditions in humans, including PCD, hydrocephalus, male infertility, chronic rhinosinusitis and chronic bronchitis. By uncovering the role of the Ccdc13–Spef1 complex in CP formation, our findings offer valuable insights into the molecular mechanisms that may underlie these disorders. Given the critical involvement of Ccdc13 and Spef1 in motile cilia function, it would be of great interest to explore whether mutations in these genes contribute to ciliopathies. Investigations into their potential as candidate genes for such conditions, especially in patient populations, could help to identify novel genetic causes of these diseases. Furthermore, understanding how Ccdc13 and Spef1 interact with other ciliopathy-related proteins could reveal deeper insights into the molecular basis of motile cilia dysfunction, with significant implications for the development of targeted clinical therapies.

## MATERIALS AND METHODS

### Fly stocks

*w^1118^* (FBal0018186) flies were used as wild-type. Cep135^c04199^ [PBac(PB)c04199, BDSC85047], Cep135^f01951^[PBac(WH)f01951] and Df(3L)BSC561 were obtained from Bloomington Drosophila Stock Center. The Cep135–GFP transgene was a gift from Prof. J. Fu (China Agricultural University). Transgenic flies of GFP–Ccdc13, Spef1a–GFP, Spef1b–GFP, Spef1b–tdTomato, CG6971–GFP, CG1571–GFP, Gas8–GFP and Rsph1–GFP, and mutant flies of *ccdc13^1^, spef1a^1^* and *spef1b^1^* were all generated in this study. All flies were kept at 25°C.

To create the transgenetic flies, cDNAs were cloned into the modified PJFRC2 vector that contained an ubiquitin promoter. These plasmids were then used to generate transgenic flies.

Mutants of *ccdc13^1^, ccdc13^2^, spef1a^1^* and *spef1b^1^* were generated by using a CRISPR/Cas9 system. To create the guide RNA (gRNA) expression plasmids, gRNAs were inserted into the PU6–BbsI–chiRNA vector through polymerase chain reaction (PCR). These plasmids were then injected into Vasa:: Cas9 embryos. Mutations were confirmed by using genomic PCR and sequencing. To confirm the mutations, genomic DNA was extracted from flies. PCR primers that flanked the gRNA target sites were used to amplify the region of DNA. The PCR products were then analysed by using gel electrophoresis to identify potential mutations, such as point mutations or large deletions. Finally, sequencing of the PCR product was performed to precisely determine the specific mutations within the gene. The mutant fly was backcrossed with *w^1118^* before use.

The primers that were used for generating and identifying the transgenetic flies and mutants are listed in Table S1.

### Immunofluorescence staining

For flies, 36–48 h after puparium formation, the testes of the pupae were dissected by using forceps and transferred to a coverslip. The coverslip was then covered with a slide and rapidly frozen in liquid nitrogen for 30 s, allowing the tissues to adhere to the slide. The tissues were first treated with methanol (–20°C) and acetone (–20°C) for fixation. Then, they were blocked with the blocking buffer (0.1% Triton X-100, 3% bovine serum albumen in phosphate buffered saline (PBS)). Next, the tissues were incubated with primary antibodies in a moisture chamber at 4°C overnight, followed by incubation with secondary antibodies for 3 h at room temperature. Images were acquired by using a laser scanning confocal microscope (Leica).

mEPCs that were grown on glass‐bottomed dishes were pre-extracted with 0.5% Triton X-100 in PBS for 30 s, followed by fixation with 4% paraformaldehyde in PBS for 15 min at room temperature. After fixation, the cells were permeabilized with 0.5% Triton X-100 in PBS for 15 min and blocked with blocking buffer (4% Bovine Serum Albumin (BSA) in Phosphate buffered saline with Triton X-100) for 1 h at room temperature. Next, the mEPCs were incubated overnight at 4°C with primary antibodies in a moisture chamber and then were washed with 1 × PBS followed by 1 h of incubation with secondary antibodies at room temperature. Finally, the mEPCs were counterstained with 4,6-diamidino-2-phenylindole (DAPI) for 20 min to label the nuclei.

Additional details on the Materials and Methods can be found in the Supplementary File.

## Supplementary Material

nwaf095_Supplemental_Files

## References

[1] Lindemann CB, Lesich KA. Flagellar and ciliary beating: the proven and the possible. J Cell Sci 2010; 123: 519–28. 10.1242/jcs.051326 20145000

[2] Shinohara K, Hamada H. Cilia in left-right symmetry breaking. Cold Spring Harb Perspect Biol 2017; 9: a028282. 10.1101/cshperspect.a028282 28213464 PMC5629998

[3] Sironen A, Shoemark A, Patel M et al. Sperm defects in primary ciliary dyskinesia and related causes of male infertility. Cell Mol Life Sci 2020; 77: 2029–48. 10.1007/s00018-019-03389-7 31781811 PMC7256033

[4] Liu H, Zheng J, Zhu L et al. Wdr47, Camsaps, and Katanin cooperate to generate ciliary central microtubules. Nat Commun 2021; 12: 5796. 10.1038/s41467-021-26058-5 34608154 PMC8490363

[5] Yuan S, Wang Z, Peng H et al. Oviductal motile cilia are essential for oocyte pickup but dispensable for sperm and embryo transport. Proc Natl Acad Sci USA 2021; 118: e2102940118. 10.1073/pnas.2102940118 34039711 PMC8179221

[6] Wallmeier J, Nielsen KG, Kuehni CE et al. Motile ciliopathies. Nat Rev Dis Primers 2020; 6: 77. 10.1038/s41572-020-0209-6 32943623

[7] Reiter JF, Leroux MR. Genes and molecular pathways underpinning ciliopathies. Nat Rev Mol Cell Biol 2017; 18: 533–47. 10.1038/nrm.2017.60 28698599 PMC5851292

[8] Ishikawa T . Axoneme structure from motile cilia. Cold Spring Harb Perspect Biol 2017; 9: a028076. 10.1101/cshperspect.a028076 27601632 PMC5204319

[9] Wirschell M, Yamamoto R, Alford L et al. Regulation of ciliary motility: conserved protein kinases and phosphatases are targeted and anchored in the ciliary axoneme. Arch Biochem Biophys 2011; 510: 93–100. 10.1016/j.abb.2011.04.003 21513695 PMC3114296

[10] Gui M, Ma M, Sze-Tu E et al. Structures of radial spokes and associated complexes important for ciliary motility. Nat Struct Mol Biol 2021; 28: 29–37. 10.1038/s41594-020-00530-0 33318703 PMC7855293

[11] Walton T, Wu H, Brown A. Structure of a microtubule-bound axonemal dynein. Nat Commun 2021; 12: 477. 10.1038/s41467-020-20735-7 33473120 PMC7817835

[12] Brokaw CJ, Kamiya R. Bending patterns of Chlamydomonas flagella: IV. Mutants with defects in inner and outer dynein arms indicate differences in dynein arm function. Cell Motil 1987; 8: 68–75. 10.1002/cm.970080110 2958145

[13] Dymek EE, Smith EF. A conserved CaM- and radial spoke associated complex mediates regulation of flagellar dynein activity. J Cell Biol 2007; 179: 515–26. 10.1083/jcb.200703107 17967944 PMC2064796

[14] Rupp G, Porter ME. A subunit of the dynein regulatory complex in Chlamydomonas is a homologue of a growth arrest-specific gene product. J Cell Biol 2003; 162: 47–57. 10.1083/jcb.200303019 12847082 PMC2172716

[15] Gui M, Wang X, Dutcher SK et al. Ciliary central apparatus structure reveals mechanisms of microtubule patterning. Nat Struct Mol Biol 2022; 29: 483–92. 10.1038/s41594-022-00770-2 35578023 PMC9930914

[16] Teves ME, Nagarkatti‐Gude DR, Zhang Z et al. Mammalian axoneme central pair complex proteins: broader roles revealed by gene knockout phenotypes. Cytoskeleton 2016; 73: 3–22. 10.1002/cm.21271 26785425 PMC4841256

[17] Carbajal‐González BI, Heuser T, Fu X et al. Conserved structural motifs in the central pair complex of eukaryotic flagella. Cytoskeleton 2013; 70: 101–20. 10.1002/cm.21094 23281266 PMC3914236

[18] Dai D, Ichikawa M, Peri K et al. Identification and mapping of central pair proteins by proteomic analysis. Biophys Physicobiol 2020; 17: 71–85. 10.2142/biophysico.BSJ-2019048 33178545 PMC7596323

[19] Han L, Rao Q, Yang R et al. Cryo-EM structure of an active central apparatus. Nat Struct Mol Biol 2022; 29: 472–82. 10.1038/s41594-022-00769-9 35578022 PMC9113940

[20] Horani A, Ferkol TW, Dutcher SK et al. Genetics and biology of primary ciliary dyskinesia. Paediatr Respir Rev 2016; 18: 18–24. 26476603 10.1016/j.prrv.2015.09.001 PMC4864047

[21] Zheng J, Liu H, Zhu L et al. Microtubule-bundling protein Spef1 enables mammalian ciliary central apparatus formation. J Mol Cell Biol 2019; 11: 67–77. 10.1093/jmcb/mjy014 30535028

[22] Fu G, Zhao L, Dymek E et al. Structural organization of the C1a-e-c supercomplex within the ciliary central apparatus. J Cell Biol 2019; 218: 4236–51. 10.1083/jcb.201906006 31672705 PMC6891083

[23] Shinohara K, Chen D, Nishida T et al. Absence of radial spokes in mouse node cilia is required for rotational movement but confers ultrastructural instability as a trade-off. Dev Cell 2015; 35: 236–46. 10.1016/j.devcel.2015.10.001 26506310

[24] McKean PG, Baines A, Vaughan S et al. Gamma-tubulin functions in the nucleation of a discrete subset of microtubules in the eukaryotic flagellum. Curr Biol 2003; 13: 598–602. 10.1016/S0960-9822(03)00174-X 12676092

[25] Zhou Q, Li Z. γ-tubulin complex in Trypanosoma brucei: molecular composition, subunit interdependence and requirement for axonemal central pair protein assembly. Mol Microbiol 2015; 98: 667–80. 10.1111/mmi.13149 26224545 PMC4636443

[26] Mottier-Pavie V, Megraw TL. Drosophila bld10 is a centriolar protein that regulates centriole, basal body, and motile cilium assembly. Mol Biol Cell 2009; 20: 2605–14. 10.1091/mbc.e08-11-1115 19321663 PMC2682601

[27] Carvalho-Santos Z, Machado P, Alvarez-Martins I et al. BLD10/CEP135 is a microtubule-associated protein that controls the formation of the flagellum central microtubule pair. Dev Cell 2012; 23: 412–24. 10.1016/j.devcel.2012.06.001 22898782

[28] Carvalho-Santos Z, Machado P, Branco P et al. Stepwise evolution of the centriole-assembly pathway. J Cell Sci 2010; 123: 1414–26. 10.1242/jcs.064931 20392737

[29] Dobbelaere J, Su TY, Erdi B et al. A phylogenetic profiling approach identifies novel ciliogenesis genes in Drosophila and C. elegans. EMBO J 2023; 42: e113616. 10.15252/embj.2023113616 37317646 PMC10425847

[30] Jiang K, Faltova L, Hua S et al. Structural basis of formation of the microtubule minus-end-regulating CAMSAP-Katanin complex. Structure 2018; 26: 375–82. 10.1016/j.str.2017.12.017 29395789

[31] Pongrakhananon V, Saito H, Hiver S et al. CAMSAP3 maintains neuronal polarity through regulation of microtubule stability. Proc Natl Acad Sci USA 2018; 115: 9750–5. 10.1073/pnas.1803875115 30190432 PMC6166842

[32] Werner ME, Mitchell JW, Putzbach W et al. Radial intercalation is regulated by the Par complex and the microtubule-stabilizing protein CLAMP/Spef1. J Cell Biol 2014; 206: 367–76. 10.1083/jcb.201312045 25070955 PMC4121976

[33] Chan SW, Fowler KJ, Choo KH et al. Spef1, a conserved novel testis protein found in mouse sperm flagella. Gene 2005; 353: 189–99. 10.1016/j.gene.2005.04.025 15979255

[34] Dong X, Lim TK, Lin Q et al. Basal body protein TbSAF1 is required for microtubule quartet anchorage to the basal bodies in Trypanosoma brucei. mBio 2020; 11: e00668–20. 10.1128/mBio.00668-20 32518185 PMC7291619

[35] Legal T, Joachimiak E, Parra M et al. Structure of the ciliary tip central pair reveals the unique role of the microtubule-seam binding protein SPEF1. bioRxiv 2024; 10.1101/2024.12.02.626492. 10.1016/j.cub.2025.06.020 PMC12258958 40651469

[36] Wu Z, Pang N, Zhang Y et al. CEP290 is essential for the initiation of ciliary transition zone assembly. PLoS Biol 2020; 18: e3001034. 10.1371/journal.pbio.3001034 33370260 PMC7793253

[37] Hou Y, Zheng S, Wu Z et al. Drosophila transition fibers are essential for IFT-dependent ciliary elongation but not basal body docking and ciliary budding. Curr Biol 2023; 33: 727–736.e6. 10.1016/j.cub.2022.12.046 36669498

[38] Riparbelli MG, Callaini G, Megraw TL. Assembly and persistence of primary cilia in dividing Drosophila spermatocytes. Dev Cell 2012; 23: 425–32. 10.1016/j.devcel.2012.05.024 22898783 PMC3422508

[39] Staples CJ, Myers KN, Beveridge RD et al. Ccdc13 is a novel human centriolar satellite protein required for ciliogenesis and genome stability. J Cell Sci 2014; 127: 2910–9. 24816561 10.1242/jcs.147785

[40] Sakai K, Miyazaki J. A transgenic mouse line that retains Cre recombinase activity in mature oocytes irrespective of the cre transgene transmission. Biochem Biophys Res Commun 1997; 237: 318–24. 10.1006/bbrc.1997.7111 9268708

[41] Sawamoto K, Wichterle H, Gonzalez-Perez O et al. New neurons follow the flow of cerebrospinal fluid in the adult brain. Science 2006; 311: 629–32. 10.1126/science.1119133 16410488

[42] Lechtreck KF, Delmotte P, Robinson ML et al. Mutations in Hydin impair ciliary motility in mice. J Cell Biol 2008; 180: 633–43. 10.1083/jcb.200710162 18250199 PMC2234243

[43] Dawe HR, Shaw MK, Farr H et al. The hydrocephalus inducing gene product, Hydin, positions axonemal central pair microtubules. BMC Biol 2007; 5: 33. 10.1186/1741-7007-5-33 17683645 PMC2048497

[44] Zhang Z, Kostetskii I, Tang W et al. Deficiency of SPAG16L causes male infertility associated with impaired sperm motility. Biol Reprod 2006; 74: 751–9. 10.1095/biolreprod.105.049254 16382026

[45] Leung MR, Sun C, Zeng J et al. Structural diversity of axonemes across mammalian motile cilia. Nature 2025; 637: 1170–7. 10.1038/s41586-024-08337-5 39743588 PMC11779644

