# Supplementary material for: Ccdc13 is essential for the assembly of ciliary central microtubules
Source: Natl Sci Rev. 2025 Mar 17;12(6):nwaf095. doi: 10.1093/nsr/nwaf095 (PMC12147715; doi:10.1093/nsr/nwaf095)
Supplement: nwaf095_Supplemental_Files [file nwaf095_supplemental_files.zip › Supplementary File 0309.docx]

**Supplementary information for**

**“Ccdc13 is essential for the assembly of ciliary central microtubules”**

**This file includes the following:**

**Figures S1 to S10**

**Tables S1 and S2**

**Legends of Movies S1 to S3**

**Supplementary Materials and Methods**


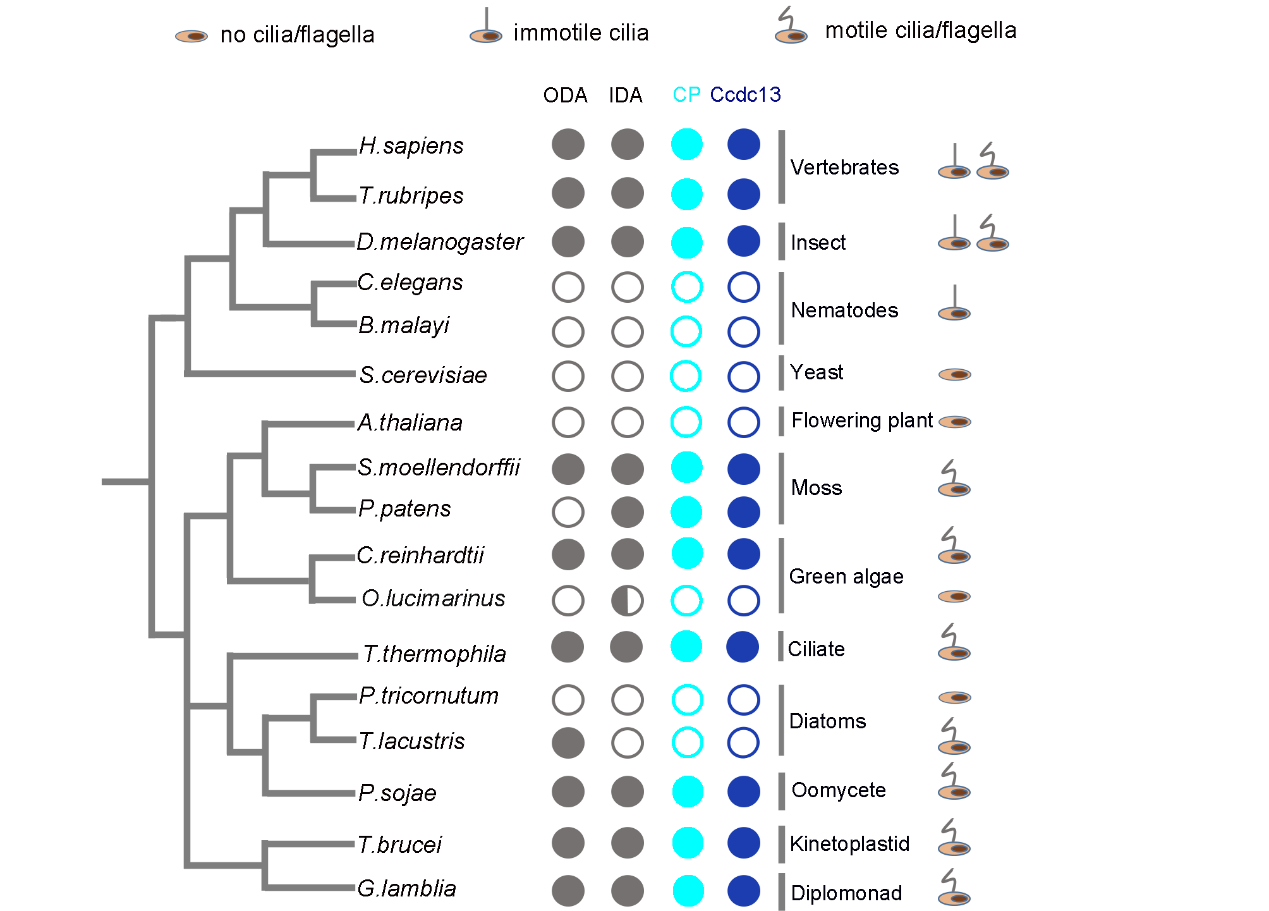
**Fig. S1**

**Supplementary Figure 1.** **Ccdc13 is evolutionarily conserved in species possessing motile cilia.** A summary of the presence of Ccdc13 orthologues in various species, as well as the outer (ODA) and inner (IDA) dynein arms, and the central pair (CP). Ccdc13 orthologues are found in species with motile cilia. Species without cilia (i.e. yeast and flowering plants) or those lacking motile cilia (i.e. nematodes) have lost Ccdc13 orthologues. For Ccdc13, filled circles indicate the existence of orthologues, as determined by the top score in reciprocal BLASTP searches, while open circles represent the absence of a homologue. Information regarding ODA and IDA is sourced from (Diggle et al., 2014), and information about the existence of CP was collected from previous studies.


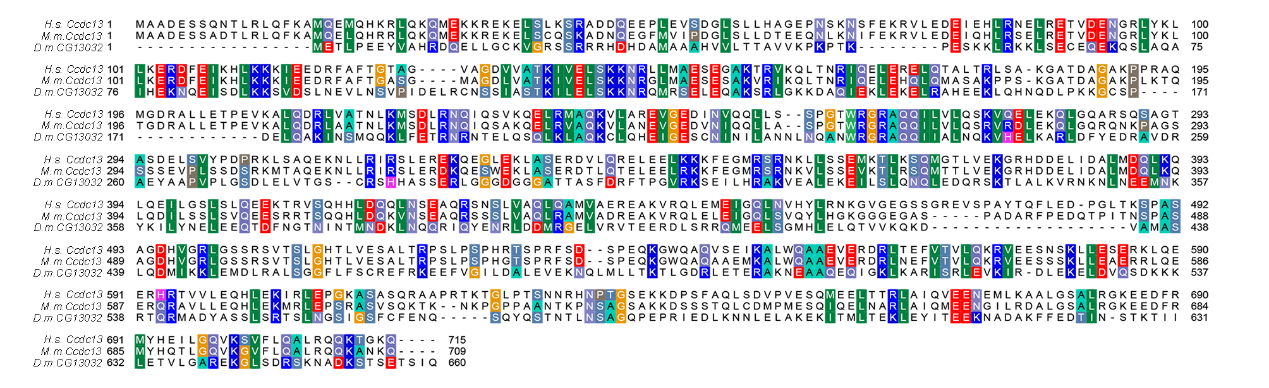
**Fig. S2**

**Supplementary Figure 2. Protein sequence alignment of human Ccdc13, mice Ccdc13 and *Drosophila* CG13032.** The protein sequence of Drosophila CG13032 was analyzed using NCBI PSI-BLAST (Position-Specific Iterated BLAST). The results revealed that mammalian Ccdc13 sequences exhibited significant similarity, with E-values better than the set threshold. Protein sequence alignment of human Ccdc13, mouse Ccdc13, and Drosophila CG13032 using ClustalW multiple alignment (BIOEDIT) indicates that Drosophila CG13032 is a homolog of the mammalian Ccdc13 protein.

**
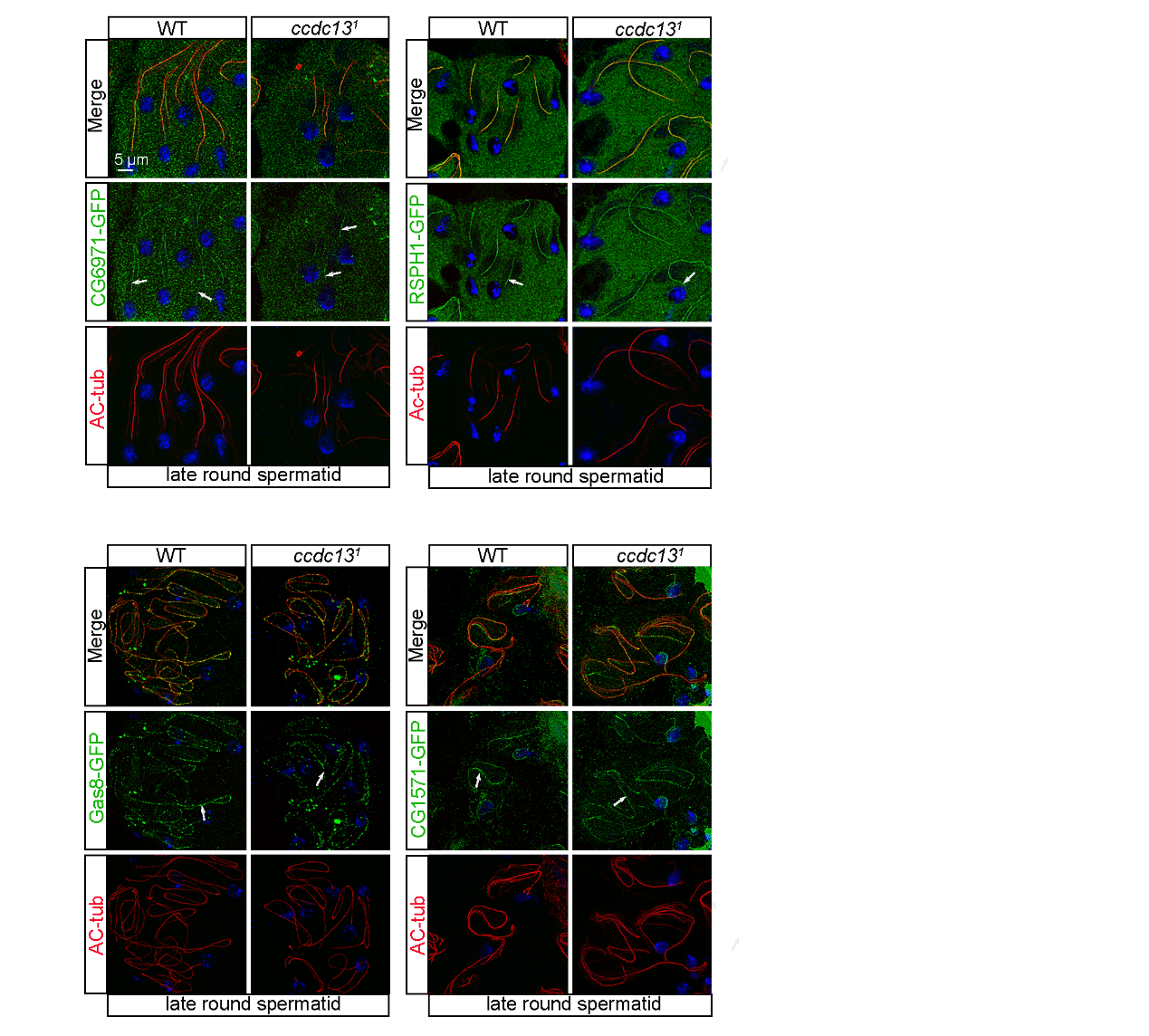
Fig. S3**

**Supplementary Figure 3.** The localization of outer dynein arm (ODA) component CG6971/Dnali1, inner dynein arms (IDA) component CG1571/Dnai2, radial spokes (RS) component Rsph1 and nexin-dynein regulatory complex (N-DRC) component Gas8 in spermatids of WT flies and *ccdc13^1^* mutants.


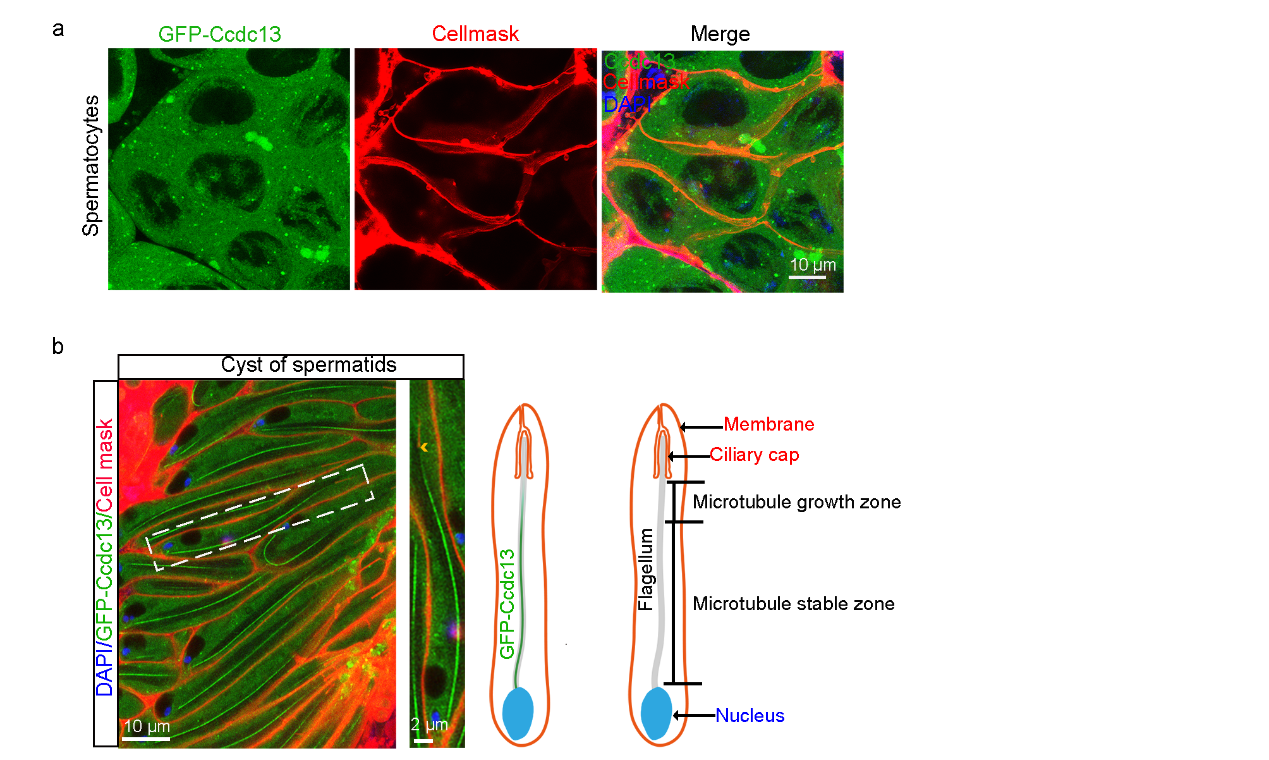
**Fig. S4**

**Supplementary Figure 4**. **Live imaging of GFP-Ccdc13 in spermatocytes and elongating spermatid of testes cys**t. (A). Live imaging of GFP-Ccdc13 in spermatocytes. (B) Live imaging of GFP-Ccdc13 in elongating spermatid of testes cyst. The plasma membrane (PM) was labeled with CellMask (red). Orange arrowhead indicates the end of flagella tip. 10 μm (full-scale images), 2 μm (zoomed-in images).


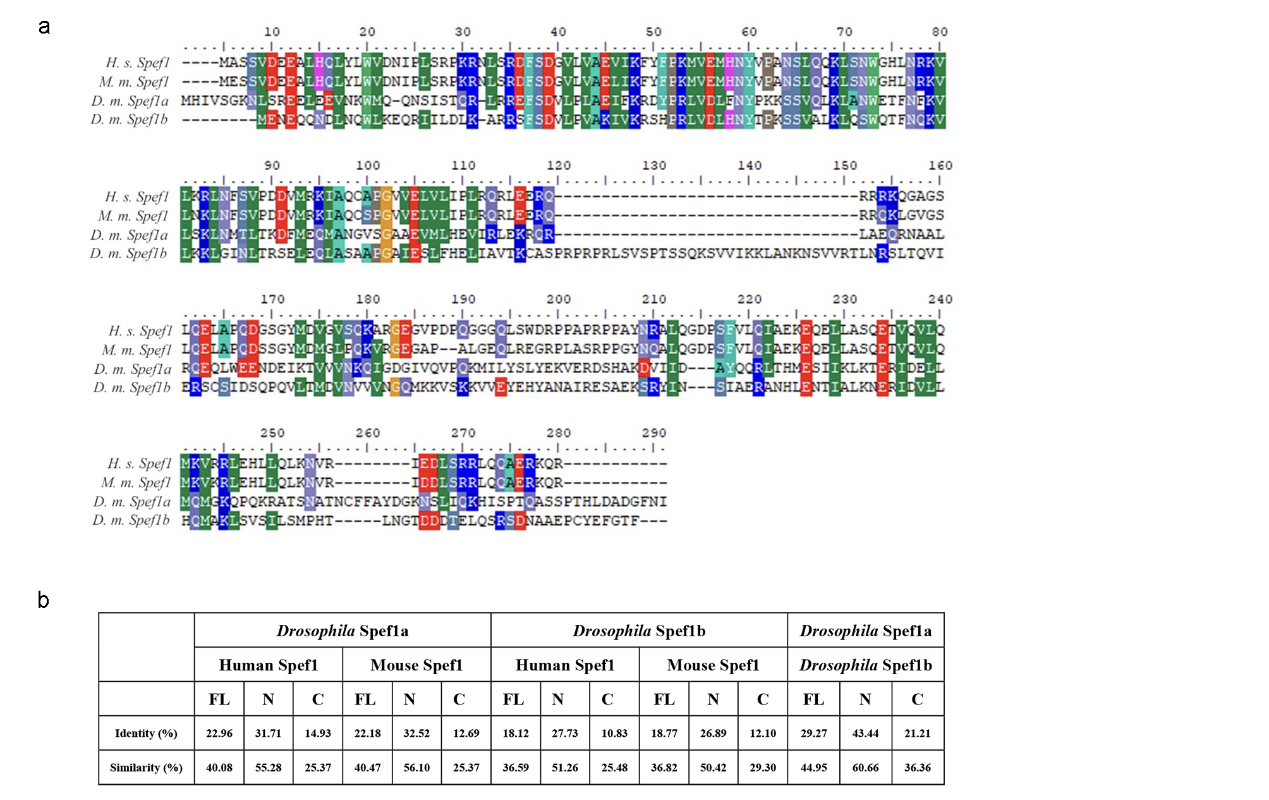
**Fig. S5**

**Supplementary Figure 5**. ***Drosophila* Ccdc13 interacts with two homologs of Spef1, namely Spef1a and Spef1b.** (A) Protein sequence alignment of human Spef1, mouse Spef1, *Drosophila* Spef1a and Spef1b. Protein sequence alignment of human Spef1, mouse Spef1, and *Drosophila* Spef1a and Spef1b using ClustalW multiple alignment (BIOEDIT) indicates that both *Drosophila* CG12395 (Spef1a) and CG16719 (Spef1b) are homologs of mammalian Spef1. (B) A table showing the sequence similarity and identity between human Spef1, and mouse Spef1, *Drosophila* Spef1a and Spef1b.

**Fig. S6**


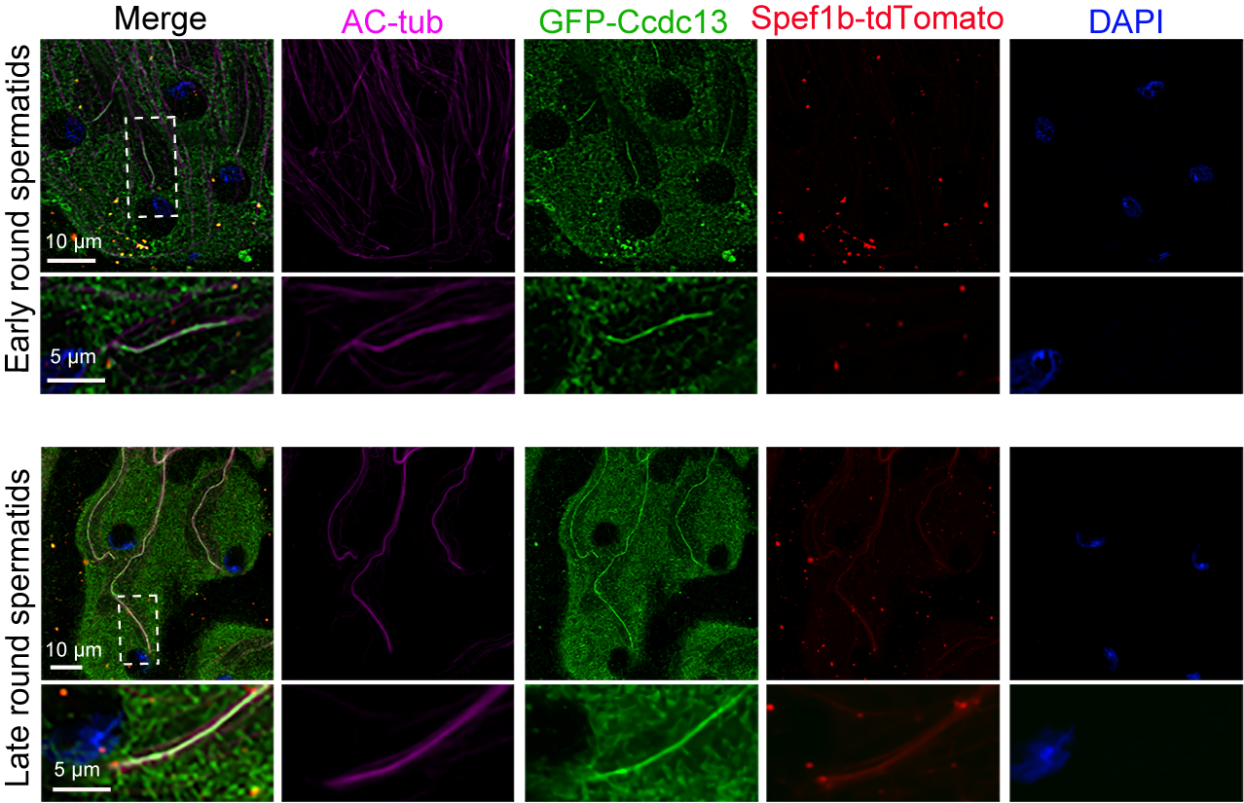


**Supplementary Figure 6. Co-localization of GFP-Ccdc13 and Spef1b-tdTomato in early and late round spermatids.**

Immunofluorescence images showing the distribution of acetylated tubulin (AC-tub, magenta), GFP-Ccdc13 (green), and Spef1b-tdTomato (red) in early and late round spermatids. DAPI (blue) marks the nuclei. In early round spermatids, GFP-Ccdc13 is present along the flagella, while Spef1b-tdTomato is not yet detectable. In late round spermatids, Spef1b-tdTomato signals become visible along the flagella, co-localizing with GFP-Ccdc13. Scale bars: 10 μm (top panels) and 5 μm (bottom panels, magnified views of dashed boxes).


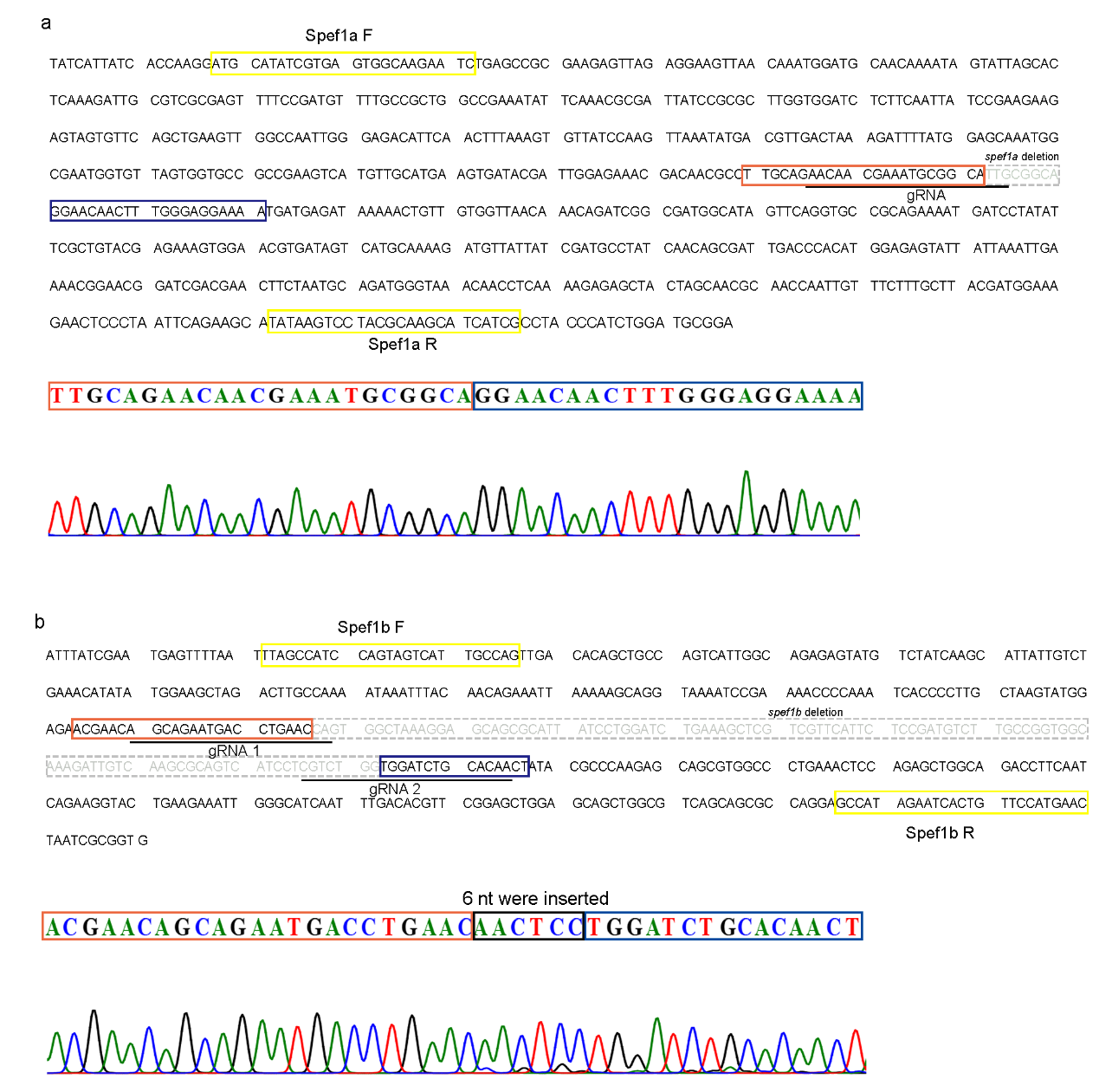
**Fig. S7**

**Supplementary Figure 7. Identification of *spef1a* and *spef1b* mutant flies.**

(A-B) Sequence confirmation of the deletion in *spef1a* and *spef1b* mutant flies. Primers used for sequence are marked with orange frames. The gRNAs sequence used for mutant generation are underlined with black. Red and blue frames label the boundary of deletion.


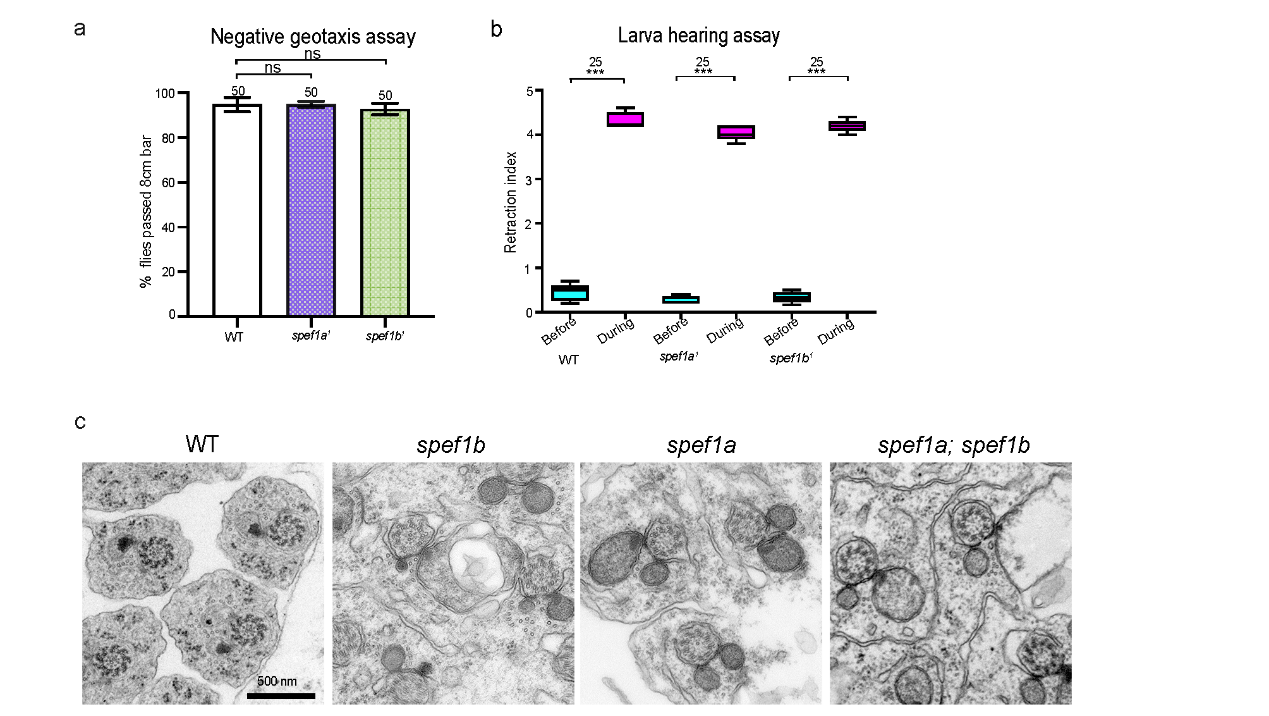
**Fig. S8**

**Supplementary Figure 8**. **Cilia-related sensory behavior assay of *spef1a* and *spef1b* mutant flies.** (A) Both *spef1a^1^* and *spef1b^1^* mutant flies showed normal negative geotaxis. (B) Both *spef1a^1^* and *spef1b^1^* mutant flies showed normal hearing ability, as determined by testing 5 larvae per group, and at least 5 groups of flies tested. Quantification results are presented as mean ± S.D. Student’s *t*-test: ns, no significance. (C) Large-scale EM images of flagellar cross-sections from WT, *spef1b^1^*, *spef1a^1^*, and *spef1a^1^;spef1b^1^* mutants.

**Fig. S9**


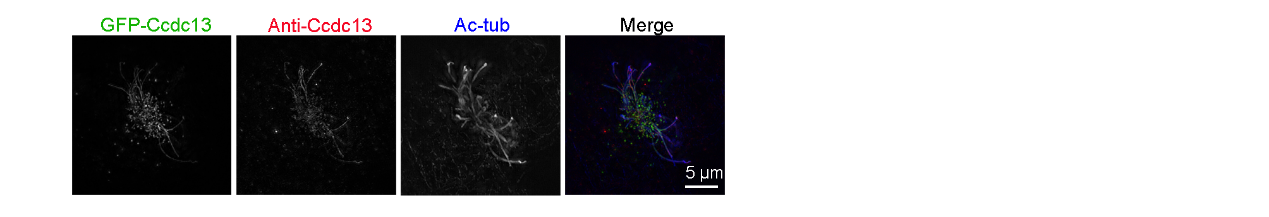


**Supplementary Figure 9. Ccdc13 localizes to the motile cilia of mEPCs.** Both GFP-Ccdc13 and Anti-Ccdc13 label the motile cilia of mEPCs. Scale bar: 5 μm

**Fig, S10**


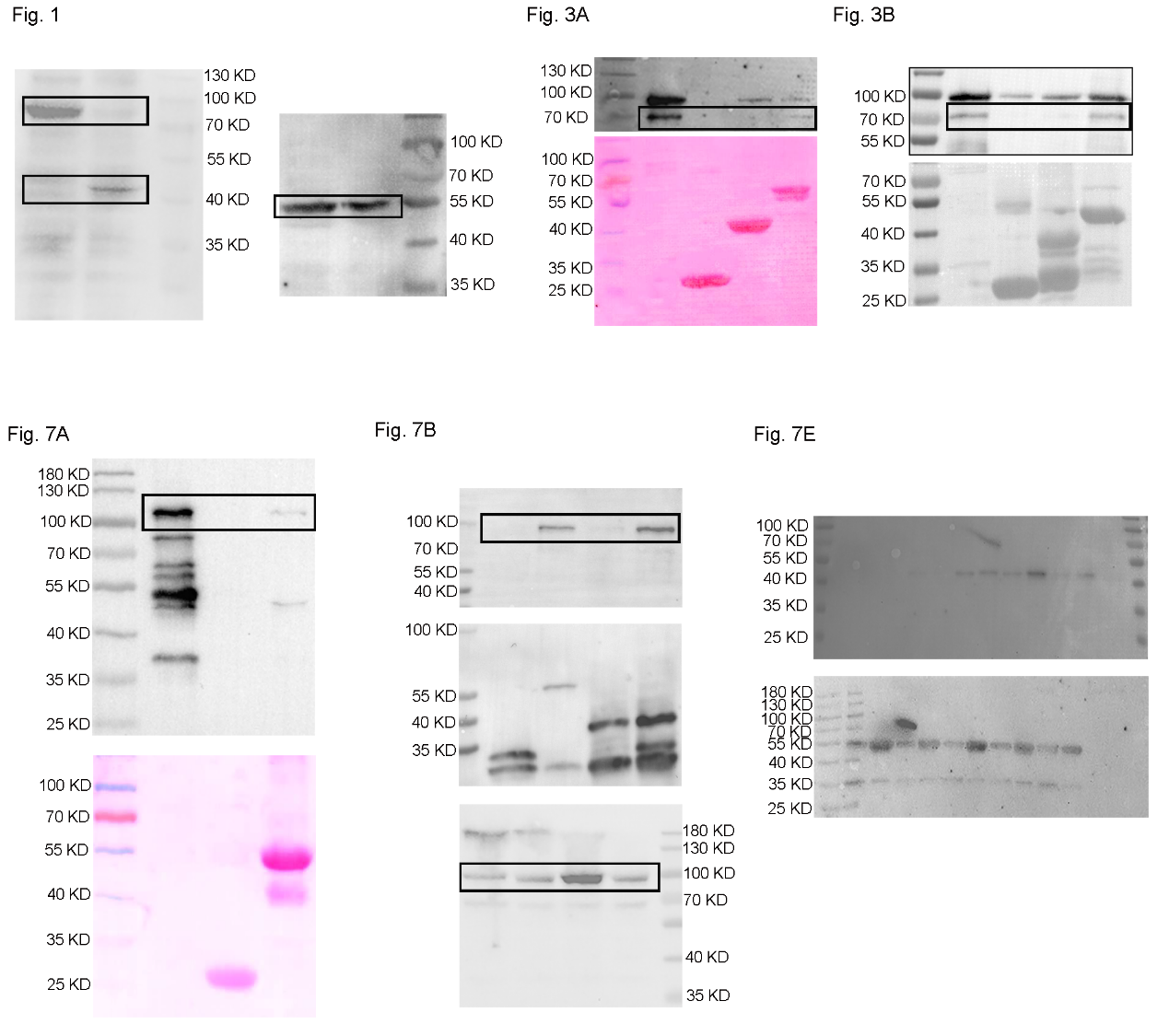


**Supplementary Figure 10. Full scans of western blots.**



**Supplementary Table 1. Primers used in this study.**


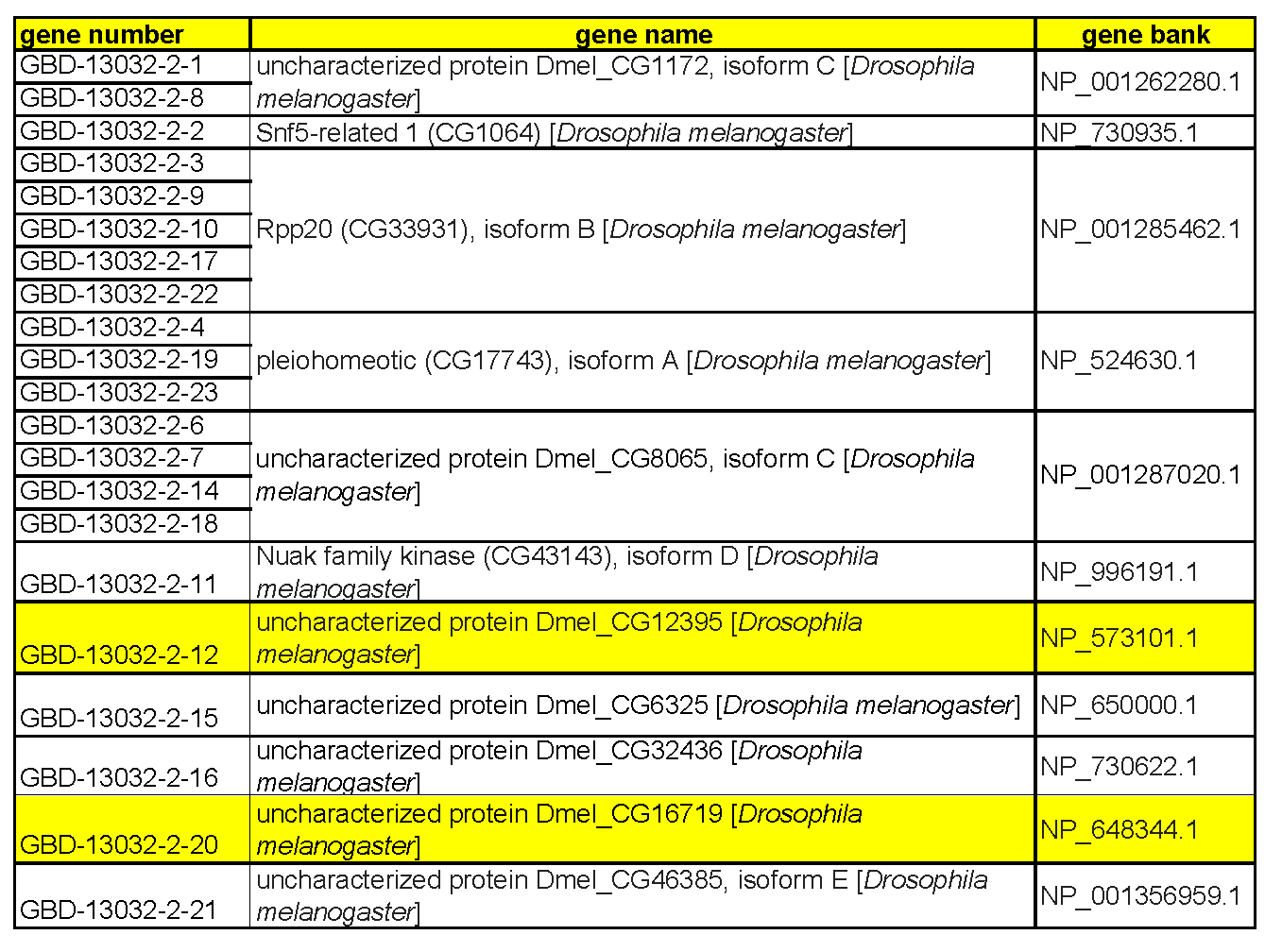
**Supplementary Table 2. Y2H screening results.**

**Supplementary Movies**

**Movie S1. Ependymal cilia beating patterns in wild type mice.**

**Movie S2. Ependymal cilia beating patterns in Ccdc13 knockout mice.**

**Movie S3. Rotational beating of ependymal cilia in *Ccdc13* mutant mice.**

**Supplementary Materials and Methods**

**Protein homology search**

To identify homologous proteins related to Ccdc13 in *Drosophila*, we used NCBI PSI-BLAST (Position-Specific Iterated BLAST) to search for human and mouse Ccdc13 homologs in *Drosophila*. This search identified CG13032 as the closest homolog in *Drosophila*. Subsequently, we performed reciprocal BLAST, using CG13032 to search human and mouse databases, which resulted in the identification of Ccdc13 homologs in both species. Protein sequence alignment of human Ccdc13, mouse Ccdc13, and *Drosophila* CG13032 was performed using ClustalW multiple alignment (BIOEDIT). The alignment confirmed that *Drosophila* CG13032 is a homolog of the mammalian Ccdc13 protein.

**Negative geotaxis assay**

For the negative geotaxis assay, 10 flies were tested as a group, with each group repeated three times, and at least five groups of flies were tested. The climbing ability of each group of flies by tapping the measuring vial to induce them to fall to the bottom, and then counted the number of flies that crossed an 8 cm high bar within a 10-second interval.

**Larval hearing assay**

For the hearing assay, five third instar larvae were tested as a group, and at least five groups of flies were tested. Larvae were intermittently stimulated with 1k Hz sound on agar plate, and the number of larvae exhibiting contractile responses on their head or body within 1 second after stimulation were quantified. Each group was replicated five times. The larval retraction response was represented by the median and interquartile range.

**Transmission electron microscopy**

For the transmission electron microscopy (TEM) assay, *Drosophila* testis, mouse sub-ependymal ventricular zone and trachea were fixed in 2.5% glutaraldehyde overnight at 4°C, washed with 1 × PBS three times, and then treated with 1% OsO_4_ for 30 min to 1h at room temperature. After extensive washing in distilled water, the sample were dehydrated using a series of ethanol concentrations (30%, 50%, 70%, 80%, 95% and 100%) and embedded in Epon 812 resin. Ultrathin sections of 70 nm were double-stained with 1% lead citrate and 2% uranyl acetate, then analyzed under a TEM (H-7650, HITACHI) with an accelerating voltage of 120 kV.

**Yeast two-hybrid (Y2H) assays**

The *Drosophila* testis cDNA library was constructed by OE Biotech (Shanghai, China). The ORF of CG13032 was amplified and cloned into the pGBKT7 vector, which was subsequently transformed into the yeast strain Y2HGold as baits. The Gold yeast two-hybrid system (Clontech) was used to screen the cDNA library with the CG13032 protein as bait. Plasmid isolation and sequencing were performed to identify the interacting protein. To validate the specific interaction between Ccdc13 and Spef1a or Spef1b, their full-length ORF were introduced into the pGBKT7 and pGADT7 vectors, respectively. Subsequently, the pGADT7 and pGBKT7 plasmids were then co-transfected into yeast strain AH109 (Takara Bio Inc.) and the interaction was verified on selective media.

**Histology hematoxylin-eosin (H&E) staining**

For H&E staining, P21 mouse brains were fixed with 4% paraformaldehyde overnight, dehydrated in ethanol, paraffin-embedded, and sectioned into 5 μm thick slices using CM1950 microtome (Leica). The sections were prepared for staining through deparaffinization in xylene, flowed by rehydration in a series of graded ethanol solutions. The sections were then stained routinely with hematoxylin and eosin for histological examination.

**Antibodies**

The following primary antibodies were used in this study: Rabbit anti-GFP (1:500, ab290, Abcam), mouse anti-GFP (1:200, 11814460001, Roche), mouse anti-Ac-tub (1:500, T7451, Sigma-Aldrich), mouse anti-γ-tubulin (1:500, T5326, Sigma-Aldrich), mouse anti-21A6 (1:200, AB528449, DSHB), Rabbit anti-Ccdc13 (1:200，PA5-61514, Thermo), Rabbit anti-Spag16 (1:200, 16883-1-AP, Proteintech), Rabbit anti-Hydin (1:500), Guinea pig anti-Hydin (1:200)[^21^](#_ENREF_21) and Chicken anti-GFP (1:200, Invitrogen). The following secondary antibodies were used: goat anti-mouse Alexa Fluor 488 (1:1000, A-11001, Invitrogen), goat anti-rabbit Alexa Fluor 594 (1:1000, A1000701, Invitrogen), goat anti-rabbit Alexa Fluor 488 (1:1000, A-11006, Invitrogen), goat anti-mouse IgG1 Alexa Fluor 488 (1:1000, A-21121, Invitrogen), goat anti-mouse Alexa Fluor 594 (1:1000, A-11007, Invitrogen), goat anti-mouse IgG2b-Alexa Fluor 647 (1:1000, A-21242, Invitrogen), donkey anti-chicken IgY-Alexa Fluor 488 (1:1000, Invitrogen), and donkey anti-guinea pig (1:1000, Jackson ImmunoResearch).

**Mice**

The mice experiments conducted in this study were carried out in strict accordance with ethical guidelines set forth by the Shanxi University, and were approved by the Committee of Scientific Research in Shanxi University. *Ccdc13^-/-^* mice were generated through the crossing the *Ccdc13^loxP/+^* mice with *Cag-Cre* mice.

Primers used for mouse genotyping are listed in Supplementary Table 1.

**Cell culture**

hTERT-RPE1，HEK293 and U2OS cell lines were maintained as an adherent monolayer in Dulbecco’s modified Eagle’s medium (DMEM) containing 10% FBS and 1% penicillin-streptomycin at 37 ̊C under a humidified atmosphere of 5% CO_2_. Multiciliated mEPCs were obtained and cultured as previously described[^4^](#_ENREF_4), with minor modifications. Briefly, telencephala from P0 mice were dissected after removing the olfactory bulbs, cerebellum, and hippocampus using sharp tweezers (Dumont,0203-5/45-PO) in cold dissection solution (161 mM NaCl, 5 mM KCl, 1 mM MgSO_4_, 3.7 mM CaCl_2_, 5 mM Hepes, and 5.5 mM Glucose, pH 7.4) under a stereo microscope. The telencephala were then digested with 1 ml of dissection solution (pre-incubated at 37°C) containing 0.2 mg/ml L-cysteine, 0.5 mM EDTA, 1 mM CaCl_2_, 1.5 mM NaOH, and 10 U/ml papain (Worthington, LS003126) for 30 min at 37°C. The cells were then gently pipetted with a P1000 tip and collected by centrifugation at 1500 rpm for 5 min at room temperature. Cells were resuspended in DMEM medium (Invitrogen, 12430047) supplemented with 10% fetal bovine serum (FBS) and 1% penicillin/streptomycin and inoculated into 25 cm^2^ laminin-coated flask (1-2 brain/flask). After culturing for 1-2 days, neurons were shaken off and removed, and the remaining cells were further cultured to ~80% confluency (usually 3-4 days) before being transferred into the wells of 35 mm laminin-coated glass-bottom dishes (Cellvis, D35-20-1.5-N). The cells were then grown in the culture medium for an additional 2 days before being maintained in serum-free medium (serum starvation) to induce multicilia formation. Multiciliated mEPCs were used for immunofluorescence staining or other assays at 5-10 days.

**Cell Transfection**

For cell transfection, cultures were plated at a density of 3×10^5^ cells per well in a 12-well plate. Plasmids encoding EGFP-tagged cDNA constructs, or both Flag- and EGFP-tagged plasmids for immunofluorescence or Co-IP assays, were transfected into the cells. Specifically, 1 μg of DNA was added to each well along with 50 μl of Lipofectamine 8000 reagent (Thermo Scientific) in OptiMEM-1 (Invitrogen). Within 48 h, cells were collected for immunofluorescence or Co-IP assays.

For mEPC Infection，GFP-tagged Ccdc13 lentiviral particles were generated by transfecting lentiviral expression plasmids, packaging plasmids pCMVdr8.9 and pMD2.VSVG into HEK293T cells. After 48 h, culture media containing the suspended lentiviral particles were centrifuged at 300 ×g for 15 min to remove cell debris. Subsequently, mEPCs were infected with lentivirus at one day before serum starvation (day -1).

**Immunoprecipitation**

To generate Spef1-GFP or Spef1-N-GFP (aa 1–181) or Spef1-C-GFP (aa 45–234), and Ccdc13-Flag co-transfected cells, HEK293T cells were co-transfected with 7.5 μg of each respective plasmid per 10 cm dish and incubated for 48 hours. The cells were then harvested and lysed with lysis buffer (10 mM Tris [pH 7.5], 150 mM NaCl, 0.5 mM EDTA, 0.5% NP-40, 1× protease inhibitor) at 4◦C for 1 h and centrifuged at 14,000 × g for 10 min at 4◦C. The supernatants were incubated with anti-GFP agarose beads (Chromotek, Planegg-Martinsried, Germany) and rotated for 4 h at 4◦C. After western blotting, the polyvinylidene fluoride (PVDF) membrane was incubated with anti-GFP antibody and either anti-CCDC13 antibody or anti-Flag antibody. Full scans of the western blots are presented in Supplementary Figure 10.

**GST pull-down assay**

For GST pull-down assays, target proteins tagged either with GST- or His-tag were overexpressed in *E. coli*, then purified from bacterial lysates using affinity beads. The fusion proteins were premixed and incubated in binding buffer (25 mM Tris-HCl [pH 7.4], 150 mM NaCl, 0.5% Triton X-100, 1 mM dithiothreitol, 10% glycerol and protease inhibitors) for 4 h at 4◦C with rotary agitation. After six washes, the beads were boiled in SDS-sample buffer and subjected to immunoblotting.

**MT Bundling Assay**

MT-bundling assay was performed as previously described[^21^](#_ENREF_21). Briefly, to obtain a concentration of 20 μM tubulin, 2 μl of tubulin stock (tubulin: rhodamine-labeled tubulin = 5:1) (Cytoskeleton, Inc.) were mixed in 2 μl of reaction buffer (80 mM PIPES, pH 6.8, 1 mM GTP, 2 mM MgCl_2_, 0.5 mM EGTA, 40% glycerol), and diluted with 40 μl of Brb80 buffer (80 mM PIPES, pH 6.8, 20 μM Taxol, 2 mM MgCl_2_, 0.5 mM EGTA) and incubated at 25°C for 30 min to assemble taxol-stabilized MTs. Spef1, Ccdc13-full and Ccdc13 truncation proteins were expressed and purified with His tags, respectively. Subsequently, the different purified proteins with the indicated concentrations were mixed with 2 μl taxol-stabilized MTs at 37°C for 10 min and then visualized on slides using Nikon microscope.

**MT pelleting Assay**

Taxol-stabilized microtubules (MTs) were assembled following the procedure outlined in the above MT bundling assay. Then 24 μl samples containing 20 μl (20μM) MTs and 4 μl varying concentrations of His-Ccdc13 (1-300) in Brb80 buffer were incubated at 37°C for 15 minutes. Subsequently, the samples underwent centrifugation at 25,000 g for 1 hour at 30°C. After centrifugation, standard western blot procedures were employed to analyze the supernatants and pellets.
